# The humanized anti-human AMHRII mAb 3C23K exerts an anti-tumor activity against human ovarian cancer through tumor-associated macrophages

**DOI:** 10.18632/oncotarget.21556

**Published:** 2017-10-07

**Authors:** Houcine Bougherara, Fariba Némati, André Nicolas, Gérald Massonnet, Martine Pugnière, Charlotte Ngô, Marie-Aude Le Frère-Belda, Alexandra Leary, Jérôme Alexandre, Didier Meseure, Jean-Marc Barret, Isabelle Navarro-Teulon, André Pèlegrin, Sergio Roman-Roman, Jean-François Prost, Emmanuel Donnadieu, Didier Decaudin

**Affiliations:** ^1^ Inserm, U1016, Institut Cochin, Paris, France; ^2^ Cnrs, UMR8104, Paris, France; ^3^ Université Paris Descartes, Sorbonne Paris Cité, Paris, France; ^4^ Laboratory of Preclinical Investigation, Translational Research Department, Institut Curie, PSL University, Paris, France; ^5^ Department of Tumor Biology, Institut Curie, Paris, France; ^6^ INSERM U896, Institut de Recherche en Cancérologie de Montpellier, Montpellier, France; ^7^ Department of Gynaecological and Oncological Surgery, Hôpital Européen Georges Pompidou, Université Paris Descartes, Assistance Publique-Hôpitaux de Paris, Paris, France; ^8^ Department of Pathology, Hôpital Européen Georges Pompidou, Université Paris Descartes, Assistance Publique-Hôpitaux de Paris, Paris, France; ^9^ Gustave Roussy Hospital, Inserm U981, Villejuif, France; ^10^ Department of Medical Oncology, Cochin Hospital, Assistance Publique-Hôpitaux de Paris, Paris, France; ^11^ GammaMabs Pharma, Centre Pierre Potier, Toulouse, France; ^12^ Department of Translational Research, Institut Curie, PSL University, Paris, France; ^13^ Department of Medical Oncology, Institut Curie, Paris, France

**Keywords:** human ovarian cancers, Müllerian hormone type II receptor, patient-derived xenografts (PDXs), chemotherapy, tumor-associated macrophages

## Abstract

Müllerian inhibiting substance, also called anti-Müllerian hormone (AMH), inhibits proliferation and induces apoptosis of AMH type II receptor-positive tumor cells, such as human ovarian cancers (OCs). On this basis, a humanized glyco-engineered monoclonal antibody (3C23K) has been developed. The aim of this study was therefore to experimentally confirm the therapeutic potential of 3C23K in human OCs. We first determined by immunofluorescence, immunohistochemistry and cytofluorometry analyses the expression of AMHRII in patient’s tumors and found that a majority (60 to 80% depending on the detection technique) of OCs were positive for this marker. We then provided evidence that the tumor stroma of OC is enriched in tumor-associated macrophages and that these cells are responsible for 3C23K-induced killing of tumor cells through ADCP and ADCC mechanisms. In addition, we showed that 3C23K reduced macrophages induced-T cells immunosuppression. Finally, we evaluated the therapeutic efficacy of 3C23K alone and in combination with a carboplatin-paclitaxel chemotherapy in a panel of OC Patient-Derived Xenografts. In those experiments, we showed that 3C23K significantly increased the proportion and the quality of chemotherapy-based *in vivo* responses. Altogether, our data support the potential interest of AMHRII targeting in human ovarian cancers and the evaluation of 3C23K in further clinical trials.

## INTRODUCTION

Ovarian cancer is the fifth most frequent cause of cancer death in women [1]. This cancer is often diagnosed at advanced stage when the disease has already spread to the upper abdomen, forming peritoneal carcinomatosis or beyond (FIGO stage III and IV, respectively) [2]. The 5-year survival rate of patients with grade III or IV ovarian cancer oscillates between 20% and 40%. Treatment of ovarian cancer usually involves a combination of reductive surgery and paclitaxel/carboplatin combined chemotherapy, but tumor relapses occur in approximately 80% of patients because of drug resistance [2, 3].

Epithelial ovarian cancers arise from coelomic epithelium and mainly from fallopian tube [4]. At the fetal stage, Mullerian structures are formed by invagination of the coelomic epithelium in the embryo and regress in the male embryo under the exposure to Mullerian inhibiting substance (MIS) which binds to Mullerian inhibiting substance type II receptor (MISRII), also called anti-Muüllerian Hormone type II Receptor (AMHRII) [5]. Recently, human Anti-Muüllerian Hormone type II Receptor (AMHRII) has been identified as a potential target for ovarian cancer therapy, on the basis that Granulosa cell tumors [6] and most of ovarian adenocarcinomas have been found to express AMHRII [7-9]. Hence, a humanized glyco-engineered monoclonal anti-AMHRII antibody, called 3C23K (Emabling^®^) and defined by an enhanced Fc effector function, has recently been developed [10].

In this study, we have investigated the therapeutic potential of 3C23K in human ovarian cancer. AMHRII expression of human tumors and Patient-Derived Xenografts (PDXs) was evaluated using various techniques, such as immunofluorescence, flow cytometry and immunohistochemistry. We first determined the immune cell populations of ovarian cancer and the effect and mechanism of action of 3C23K on these cells, and particularly on Tumor-Associated Macrophages (TAM). We finally studied the *in vivo* activity of 3C23K alone and in combination with carboplatin-paclitaxel in various ovarian cancer PDXs. Altogether, due to a significant AMHRII expression in ovarian cancers and specific immune-dependent anti-tumor activity, our results support the evaluation of 3C23K in ovarian cancer patients in clinical trials.

## RESULTS

### AMHRII receptor is expressed in a high proportion of ovarian cancer patient’s tumors and PDXs

**Figure 1:**
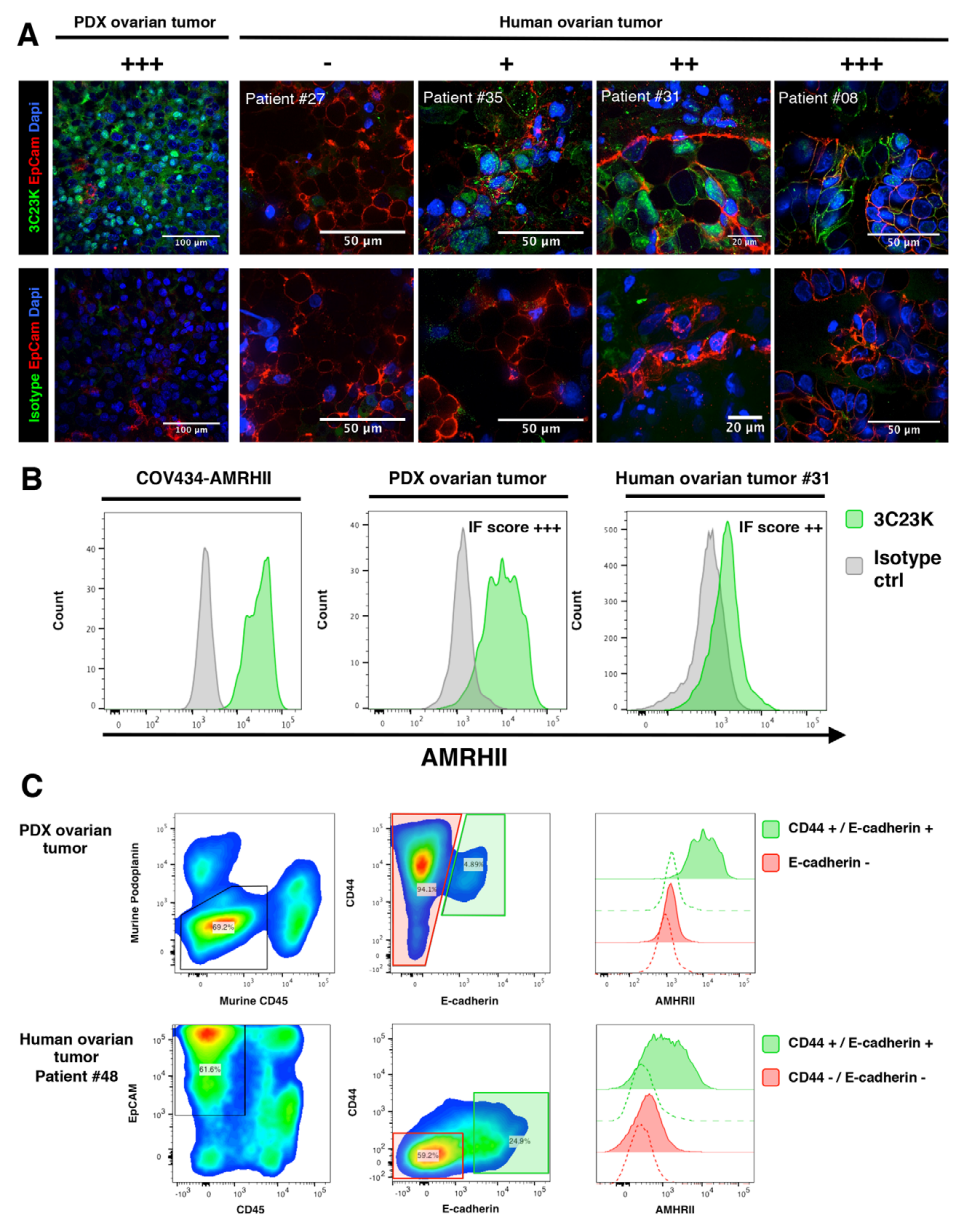
Immunofluorecence determination of AMHRII expression: AMHRII is heterogeneously expressed in human ovarian cancers and stained by the glycoengineered anti-human AMHRII humanized mAb 3C23K **(A)** Representative multiplexed immunofluorescence microphotographs showing AMHRII detection by the AlexaFluor488 conjugated 3C23K mAb (green fluorescence channel, upper panels) compared to the AlexaFluor488 conjugated R565 isotype control mAb (green fluorescence channel, lower panels) in the PDX ovarian tumor (Ov54) and in four fresh human ovarian malignant biopsies, ranging from negative to high AMHRII expressing tumors. A qualitative score (- to +++) was attributed to each sample. Tumor nests were identified by EpCAM expression (red fluorescence channel). Nuclei were stained with DAPI (blue fluorescence channel). **(B)** AMHRII detection by the AlexaFluor488 conjugated 3C23K mAb in the reference cell line COV434-AMHRII (left panel), the OV54 PDX human ovarian tumor model (middle panel), and a human ovarian tumor biopsy (right panel) after tissue digestion and multi-parametric flow cytometry analysis. Plots were gated on CD45 negative cells, namely tumor cells and stroma cells. **(C)** Multiplex analysis of human ovarian tumor by flow cytometry. High expression of AMHRII, as detected by the 3C23K, is predominantly found within CD44+ E-Cadherin+ double positive tumor cell subsets in both PDX ovarian tumors (upper panels) and the human ovarian tumor #48 (lower panels, representative of 3 patients). On human dissociated biopsies, tumor global population was defined as positive for EpCAM and negative for CD45. On PDX ovarian tumor, tumor cells were defined as negative for both murine podoplanin, a stromal cell marker, and CD45.

AMHRII expression was assessed on ovarian tumor samples using immunofluorescence (IF), immunohistochemistry (IHC), or Flow cytometry (FC). The AMHRII expression was first assessed by IF on 34 fresh non-fixed human ovarian tumor biopsies and 5 PDXs. Tumor cells were identified by EpCAM expression. Human tumor expression of AMHRII was confirmed by colocalization of EpCAM and AMHRII positive cells (except for PDX OV54 which lacks EpCAM expression). Representative pictures are shown in Figure 1A. Overall 71% (24/34) of human tumor samples were found to express AMHRII at membranous level. Distribution, according to intensity was as follow (+++ n=2), (++ n=10), (+ n=12) (Supplementary Table 1). This IF scoring of AMHRII expression was confirmed in a patient’s biopsy (#31), a PDX model (OV54), and the human GCT cell line COV434-AMHRII by FC detection of AMHRII (Figure 1B). Only a subset of tumor cells express AMHRII with an average of 12.6% (Figure 1A). Interestingly, AMHRII expression was more pronounced in the CD44/E-Cadherin positive tumor subset, as observed by multiplexed FC, both in the PDX models and human tumor samples (Figure 1C). Among the 5 PDXs models in which AMHRII membrane expression was determined, OV54 displayed the higher expression whereas OV16, OV42, OV25, and OV21 displayed a moderate expression (Supplementary Figure 1). Finally, CD45 and EpCAM immunostaining was performed in four PDXs (OV8, OV42, OV54, and OV57), and confirmed that AMHRII-positive cells are epithelial, in particular the OV54 model (Supplementary Figure 2).

IHC study was performed in 26 other ovarian patient’s tumors and their corresponding PDXs, and the two xenografts obtained from the COV434-wt and -AMHRII cell lines. Positive control included two granulosa ovarian cancers (Supplementary Figure 3). When patient’s tumors and their corresponding PDXs were compared, we observed a similar membrane score in 21 couples (81%), a higher score in patient’s tumors in 3 cases (11%), and a higher score in PDXs in 2 couples (8%). When IF and IHC studies can be compared, i.e. in OV54 PDX at various *in vivo* passages, it appeared that cell membrane IHC immunostaining was lower than IF determination, suggesting that tumor fixation may have an impact on the intensity of the immunostaining; however, we did not observe discrepancy regarding the proportion of AMHRII-positive tumor cells.

**Figure 2:**
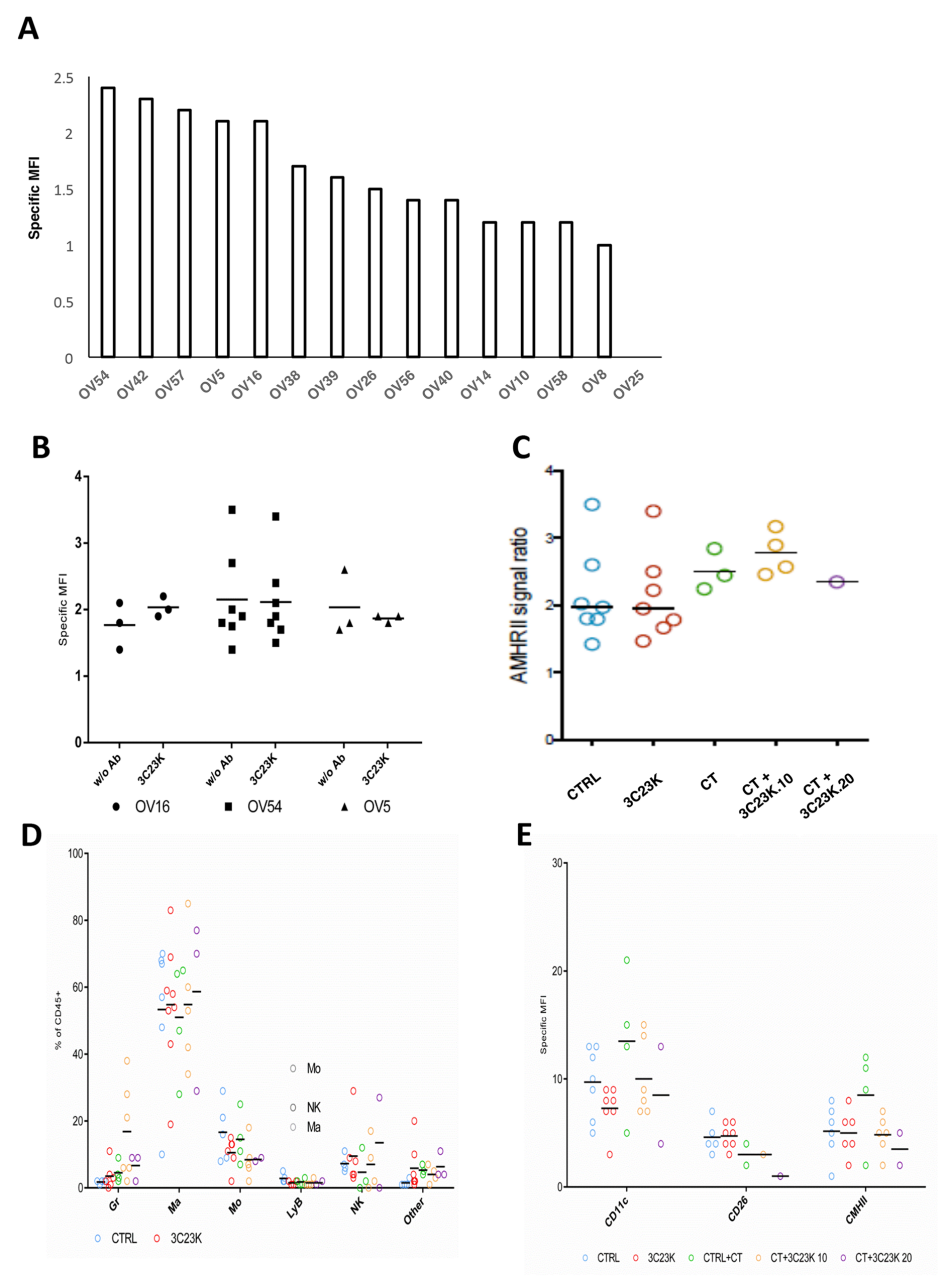
Cytrofluometric analysis of ovarian cancer cell lines and PDXs **(A)** AMHRII expression of various ovarian cancer cell lines and PDXs. The expression of receptor was expressed by its specific Mean Fluorescent Intensity (MFI: arbitrary unit of signal to noise ratio from measured fluorescence) stated as the Geometric Mean (adapted for logarithmic distributions) of majority human population stained with 3C23K weighted with MFI of this population stained with R565 (non-specific signal). **(B) (C)** Expression of AMHRII in three OC PDXs at time sacrifice after *in vivo* administration of 3C23K and/or chemotherapy (CT) (carboplatin + paclitaxel). **(D)** Immune cell component composition in the OV54 OC PDX after various *in vivo* treatments. **(E)** M1/M2 macrophage proportion in the OV54 OC PDX after various *in vivo* treatments.

AMHRII expression was also assessed by FC in 15 OC PDXs. The results are presented in the Figure 2A and in the Supplementary Figure 4, and showed a first group of tested tumors whose MFI (arbitrary unit of signal to noise ratio from measured fluorescence) was greater than 2 (OV54, OV42, OV57, OV5, and OV16), a group that could be considered as strictly positive-AMHRII tumors; a second group composed of 5 PDX models (OV38, OV39, OV26, OV56, and OV40) with a MFI between 1,2 and 2 corresponding to moderate AMHRII expression tumors; and a third group of 5 xenografts (OV14, OV10, OV58, OV8, and OV25) with a specific MFI lower than 1.2 corresponding to negative AMHRII specimens.

The number of AMHRII binding site per cell (equivalent to the cell surface number of receptor) was quantified by FC in 4 tumor patient’s biopsies including 1 IF negative and 3 IF positives for AMHRII. The human tumor that we identified as negative by IF expressed a barely detectable number of receptors (Antigen Binding Site = 350), while positive tumors displayed a significant number of receptors with an average of 48000 compared to the cell line COV434-AMHRII expressing an average of 90000 receptors.

### Human ovarian tumors and PDXs are defined by a predominant infiltration of tumor-associated macrophages

**Figure 3:**
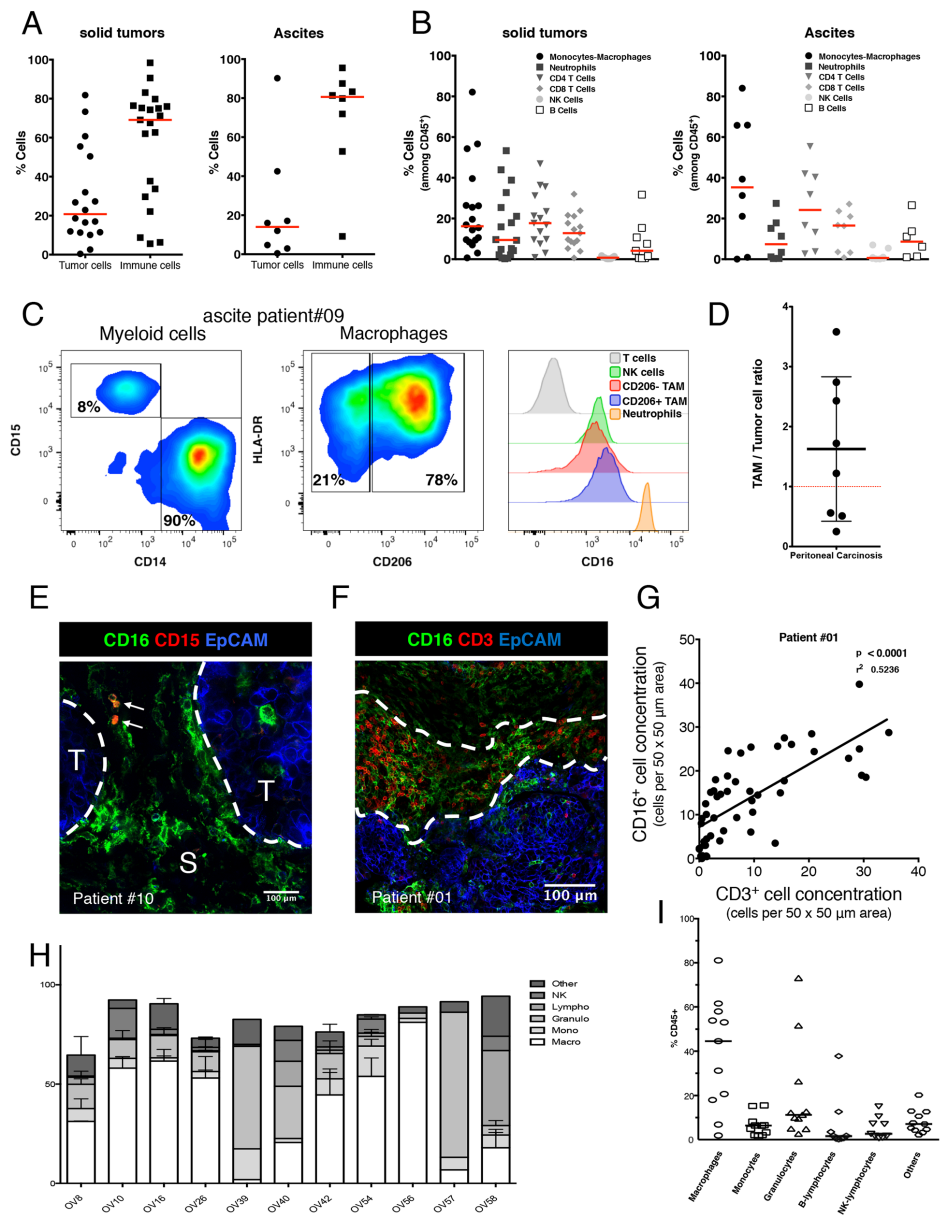
Phenotypic characterization of human ovarian tumors infiltrating immune cells **(A)** Relative quantification of tumor cells (defined as EpCAM+/CD45- cells) compare to immune cells (defined as EpCAM-/CD45+ cells) in 21 solid tumors (left panel) and 8 ascites (right panel) by multiplexed flow cytometry. Results are analyzed after doublet and dead cells exclusion. Data are expressed as the cell percentage within the singlet living population. Each dot represents a different patient and the median is shown (red bar). Both solid tumor and ascites are most frequently composed of a high proportion of immune cells. **(B)** Immune cell subset characterization in 21 solid tumors (left panel) and 8 ascites (right panel) by multiplexed flow cytometry. Results are analyzed after doublet and dead cells exclusion. Data are expressed as the cell percentage within the singlet living immune cell population (defined as EpCAM-/CD45+ cells). Each dot represents a different patient and the median is shown (red bar). Immune infiltrate of both solid tumor and ascites is composed of two major subsets: Macrophages (TAMs) and T cells (TILs). While NK cells remain scarce. **(C)** Immune cells composing a representative tumor sample (ascites from patient #09) were analyzed by flow cytometry for TAM phenotype (left and middle panels) and for surface expression of Fc gamma RIII (right panel). **(D)** Represented E/T ratio as the total CD16 positive immune cells versus tumor cells was measured by flow cytometry in 8 OC patients. The average Effector/Target ratio is 1.8/1(black bar). **(E-G)** Distribution of TAMs and TILs in the tumor microenvironment: (E) Representative multiplexed immunofluorescence microphotographs showing CD16 expression (green fluorescence channel) compared to CD15 expression (red fluorescence channel) in a fresh human ovarian malignant biopsy (patient #10) Tumor nests were identified by EpCAM expression (T, blue fluorescence channel). S, Stroma. CD16 expressing TAMs are mostly in direct contact with the tumor/stroma border (white dashed lines) and are even able to infiltrate into the core of the tumor nest while neutrophils (CD15+/CD16+ double positive cells, white arrows) are rarely found and more localized in distal stromal areas. Representative of 16 OC patient’s biopsies. (F) Representative multiplexed immunofluorescence microphotographs with CD16 expressing cells including TAMs (green fluorescence channel) compared to CD3 expressing T cells (red fluorescence channel) in a fresh human ovarian malignant biopsy (patient #01). Tumor nest is identified by EpCAM expression (T, blue fluorescence channel). (G) Positive correlation between TAMs and TILs concentration expressed as the number of cells per 50 x 50 μm area. TAMs and TILs tend to localize in the same peritumoral areas fostering cell-to-cell contact between those two populations. **(H)(I)** Proportion of immmune cells present (CD45+ cells) in various OC PDXs.

Immune cells infiltrating solid tumors (n=21) and ascites (n=8) derived from OC patients were phenotypically characterized by multiplexed FC. Immune cells (CD45+) were the predominant cells versus tumor cells (EpCAM+) both in solid tumors and ascites but with large variability between patients. In solid tumors the proportion of immune cells ranges from 5 to 98% with a median value of 69.1% (Figure 3A). TAMs (CD11b+/CD3-, CD14+/CD15- cell subset) represent 16.2% and 35.3% of all immune cells (CD45+) in solid tumor and ascites, respectively. TILs (CD11b-/CD3+, CD4+ and CD8+ cell subset) represent 30.5% and 40.7% of all immune cells (CD45+) in solid tumor and ascites, respectively. Neutrophils and B cells represent less than 10 % of all immune cells in solid tumor and ascites. NK cells represent less than 1% of all immune cells in both solid tumor and ascites (Figure 3B).

We also analyzed the distribution of CD16 expression within tumor infiltrating immune cells. A more specific immunophenotyping revealed that most of TAMs express the CD206 marker of M2-like macrophages. These cells retain a high expression of the Fc gamma III Receptor (CD16) compared to NK cells and CD206 negative M1 polarized TAMs (Figure 3C). These data demonstrate that TAMs are the most frequent Fc gamma III receptor bearing effector cells in the tumor microenvironment of OC patient while NK cells remain scarce. Another parameter that could influence the therapeutic response of anti-tumor mAb-based immunotherapy is the “Effector”/”Target” (E/T) ratio. This parameter has been calculated as CD16 expressing TAM cells versus EpCAM+ tumor cells measured by flow cytometry in 8 OC patients. E/T was 1.8/1 (Figure 3D), whilst in ascites, this E/T ratio was 3.1/1 (Figure 3A-3B).

Besides the quantity of mAb effector cells infiltrating the tumors, the localization within tumor microenvironment is a crucial feature to consider for optimal anti-tumor responses with mAbs. Multiplexed IF exploration of 16 ovarian tumors using a spinning disk confocal illustrated that CD16+ TAMs which mostly express CD206 are localized in close vicinity of tumor nests while CD15+ neutrophils are more often found in distal stromal areas (Figure 3E and Supplementary Figure 5). Because of their localization, TAMs might induce a mAb dependent anti-tumor effect via CD16. Furthermore, TAMs tend to accumulate in peritumoral areas where TILs are also concentrated (Figure 3F-3G). This suggests that TAMs are potentially promoting immunosuppression of T cell anti-tumor response by cell-to-cell contact with TILs.

We have also determined the immune cell populations present in 10 models of ovarian cancer PDX (Figure 3H-3I). We have observed that 7 out of 10 models were characterized by the presence of mainly macrophages (OV8, OV10, OV16, OV26, OV42, OV54, and OV56), 2 by mainly granulocytes (OV39 and OV57), one by mainly B-cells (OV58). The models OV40 and OV42 displayed a similar proportion of macrophages and granulocytes. Overall, macrophages constitute the predominant immune cell population found in ovarian cancer solid tumors, ascites and PDXs.

### The 3C23K antibody displayed a binding profile to Fcγ receptors similar to that described with other low-fucose IgG1

Binding profile of 3C23K to human Fcγ receptors was determined by Surface Plasmon Resonance (SPR) (Supplementary Table 2). Indeed, as other glyco-engineered antibodies [11, 12], 3C23K was expected to display improved affinities towards the different classes of Fcγ receptors, with especially a substantial increase in CD16a affinity. Hence, affinity profile of 3C23K for human receptors was classified as follows: CD64 (0.2nM) > CD16a (1.3nM) > CD16b (49nM) > CD32a (120nM) > CD32b/c (459nM).

In order to anticipate interpretation of *in vivo* experiments, the binding profile of 3C23K to murine homologs of Fcγ receptors was also determined under the same experimental conditions. Affinity measures with 3C23K for most murine homologs of Fcγ receptors was approximately 10 to 100-fold inferior to those observed with the human receptors. The only affinity conserved from human to murine homologs concerned the CD16a (or hFcγRIIIA) receptor and its murine homolog, mFcγRIV, with optimal Kd values of 1.3 and 2.1nM, respectively.

### 3C23K elicits ADCC and ADCP of ovarian tumors cells *in vitro* and *ex vivo*

**Figure 4:**
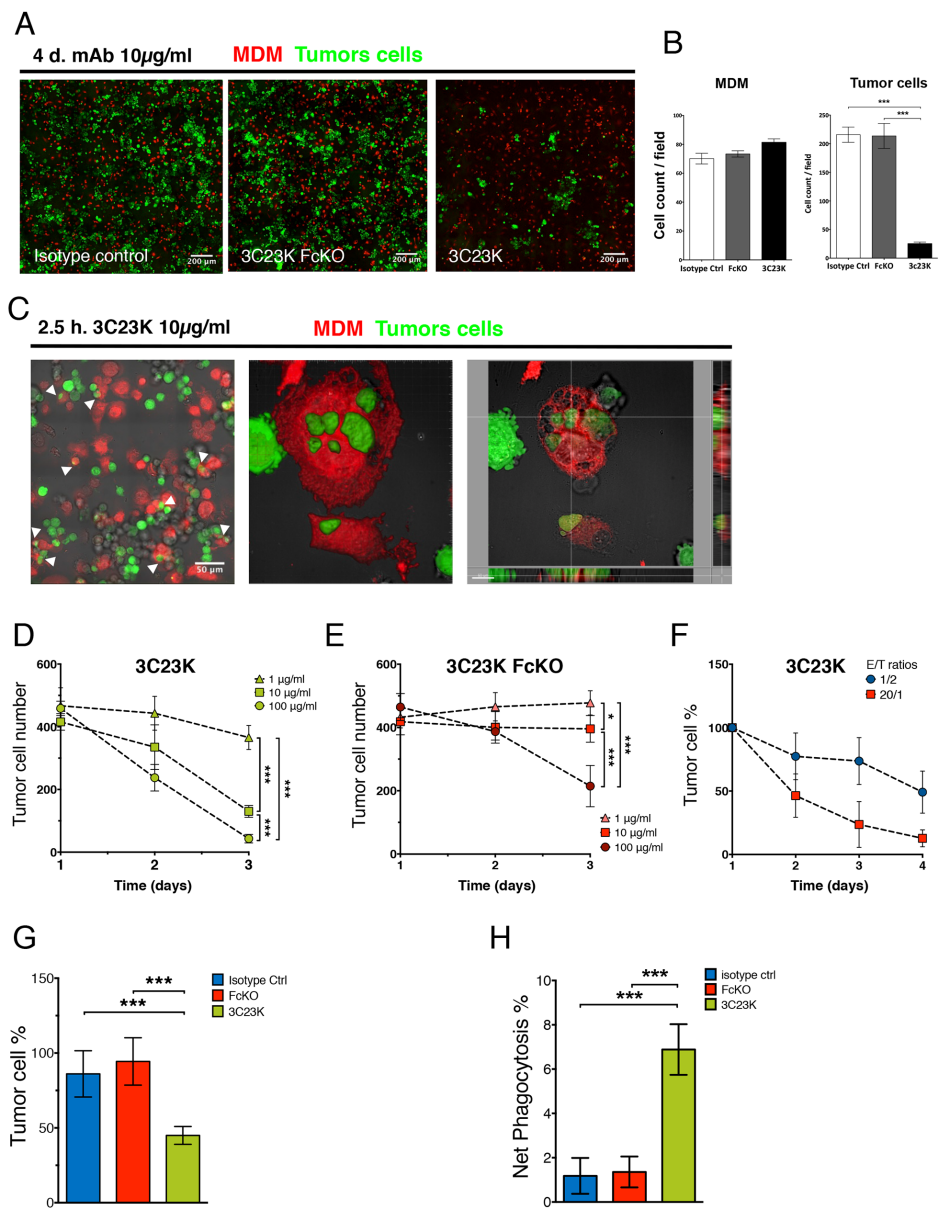
3C23K eliminates ovarian tumors cells by directing TAM to induce ADCC and ADCP *in vitro* and ex vivo **(A)** Representative IF microphotographs of MDM2/COV434-AMHRII co-culture after 4 days of treatment with either the irrelevant mAb R565, the anti-AMHRII FcKO or the anti-AMHRII 3C23K. Cell Trace Violet^®^ stained MDM2 are in red and CMFDA^®^ stained COV434-AMHRII cells are in green. **(B)** Quantification of MDM2 and COV434-AMMHRII tumor cells after 4days of treatment with either the irrelevant mAb R565, the anti-AMHRII FcKO or the anti-AMHRII 3C23K. Data are expressed as the cell count per field of view (FOV) +/- Standard Deviation. A student t-test was performed. ^***^ p < 0.005. **(C)** Representative IF microphotographs of MDM2/COV434-AMHRII co-culture after 2.5h of treatment with the anti-AMHRII mAb 3C23K. Cell Trace Violet^®^ stained MDM2 are in red and CMFDA^®^ stained COV434-AMHRII cells are in green. (Left panel) White arrows indicate phagocytosis events, (Middle panel) reconstituted 3D volume view, (Right panel) reconstituted 3D section view. 3C23K targeted tumor cells are engulfed by MDM2 macrophages. **(D-F)** MDM mediated ADCC *in vitro* assay by quantification of viable COV434-AMHRII tumor cells during 4 days of treatment. (D-E) ADCC dose responses. MAb concentrations = 1 (triangles), 10 (squares), and 100 μg/ml (circles), E/T ratio = 1/2. Data are expressed as the numbered tumor cell +/- Standard Deviation. P-values ^*^ < 0.05, ^***^ < 0.001. (Triplicates). (D) 3C23K mAb dose response. (E) 3C23K-FcKO mAb dose response **(F)** Kinetic response with varying E/T ratios. E/T ratio = 1/2 (blue circles) or 20/1 (red squares), mAb concentration = 10μg/ml. Data are expressed as the tumor cell percentage normalized at day1 +/- Standard Deviation. **(G-H)** *Ex vivo* mAb assays of mAb anti-tumor response with total primary cells from OC patient’s ascites (n=2) co-cultured with COV434-AMHRII tumor cells treated with either the irrelevant mAb R565 (blue bars), the anti-AMHRII FcKO (red bars) or the anti-AMHRII 3C23K (green bars). (G) ADCC *ex vivo* assay by quantification of viable COV434-AMHRII tumor cells after 24 hours of mAb treatment. Data are expressed as the tumor cell percentage +/- Standard Deviation. P-values ^***^ < 0.001. (H) ADCP *ex vivo* assay by quantification of COV434-AMHRII tumor cells engulfed by primary macrophages from OC patient ascites after 24 hours of mAb treatment. Data are expressed as the CMFDA stained tumor cell percentage into the CD14 positive cell fraction of total primary ascites cells +/- Standard Deviation. P-values ^***^ < 0.001.

We tested the anti-tumor activity of 3C23K in *in vitro* assays with COV434-AMHRII used as tumor cells and monocyte-derived type 2 macrophages (MDM2) used as effector cells (Supplementary Figure 6). After 4 days of exposure, 3C23K induces a strong and significant reduction of tumor cell numbers, as assessed by IF, whereas no effects were observed with control mAbs (Figure 4A and 4B). The mechanism of ADCC was then investigated. Using high resolution confocal microscopy, we evidenced phagocytosis of tumor cells by MDM2 characterized by the uptake of tumor cells by macrophages and the establishment of larges vacuoles referred to as phagosomes (Figure 4C). Notably, no reduction of tumor cell number and no induction of phagocytosis were observed with the Fc-mutated counterpart of 3C23K. The study of mAbs treatment (with various concentrations of antibody and various kinetics points) revealed that only 3C23K is able to significantly induce ADCC after 2-3 days of incubation at E/T ratio (1/2) even lower to those measured in OC (∼2/1) (Figure 3D), compared to controls as measured by IF quantification (Figure 4D-4F). Whereas 3C23K induced ADCC in a dose-dependent manner with some effects observed at concentrations as low as 1 μg/ml, a 3C23K variant bearing both G236R/L328R mutations on Fc region that abolish interaction with CD16, as described by Horton et al. (2008) [13], only induced ADCC at 100 μg/ml, a very high concentration which as not used thereafter (Figure 4D and 4E). Those results demonstrate that 3C23K induces ADCC and that ADCP activity is implicated in this process.

Similar experiments were then performed with ascites from two OC patients. To this end, 3C23K opsonized COV434-AMHRII target cells, stained with the green dye CMFDA, were co-cultured with total ascites cells. As shown in Figure 4G, the number of tumor cells was statistically and significantly decreased from the first day of treatment with 3C23K. Anti-tumor effects were neither seen in presence of the irrelevant mAb R565 nor in the presence of the FcKO anti-AMHRII (Figure 4G). We have also assessed the capacity of 3C23K to induce ADCP by determining the percentage of CMFDA (COV434-AMHRII)/CD14 double positive cells among total COV434-AMHRII cells (CMFDA simple positive + CMFDA/CD14 double positive). ADCP was measurable from the first day of treatment (Figure 4H), consistent with a rapid process, mostly occurring during the first hours of incubation. Together these data document that 3C23K specifically triggers ADCC and ADCP mediated by tumor-associated effector cells from OC patients. Of note, 3C23K did not induce significant changes in total CD14+ Macrophages and TAMs (CD206+/CD163+ cells) (Supplementary Figure 7A and 7B).

### 3C23K mAb reduces macrophages induced-T cells immunosuppression

**Figure 5:**
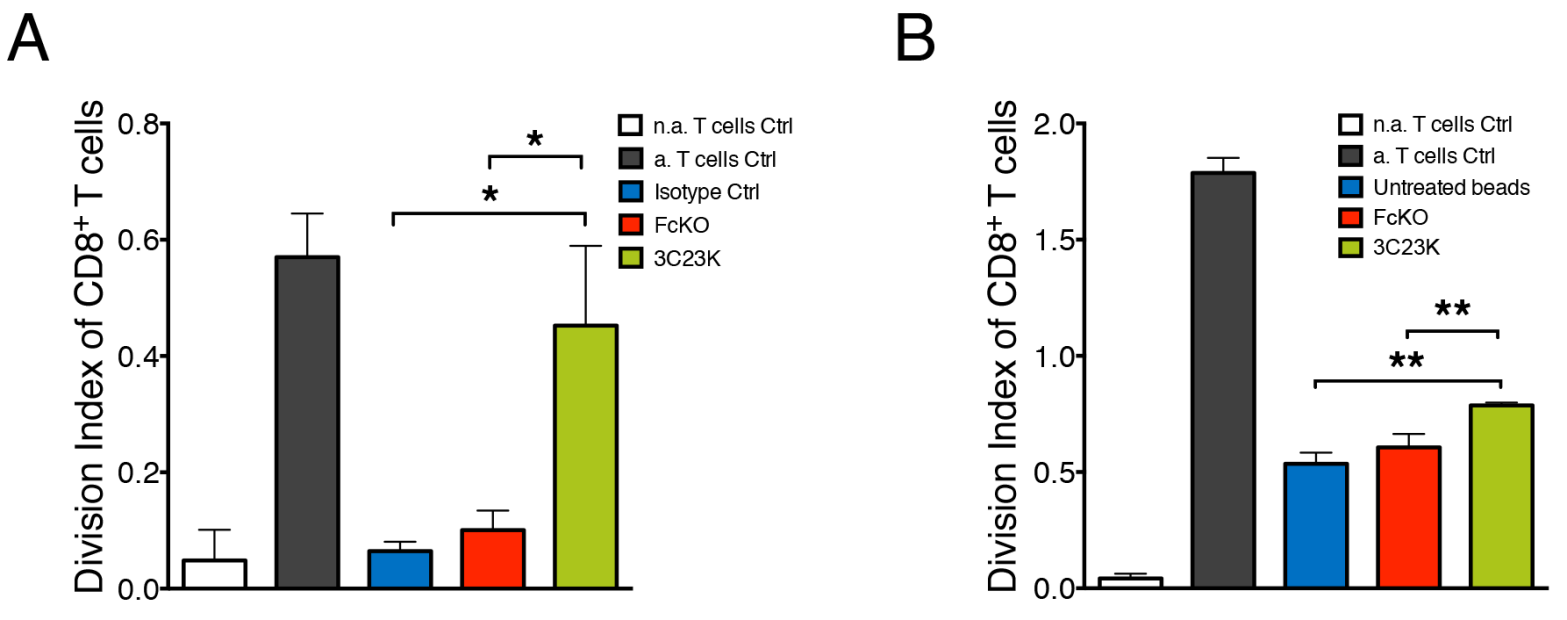
3C23K mAb reduces macrophages induced-T cells immunosuppression **(A)** Measure of PBT proliferation co-cultured with MDM2 macrophages targeting COV434-AMHRII tumor cells. MDM2 were challenged with COV434-AMHRII opsonized with either the irrelevant mAb R565 (isotype Ctrl), the anti-AMHRII FcKO or the anti-AMHRII 3C23K for 4 days prior the co-culture for an additional 4 days with anti-CD3/CD28 pre-activated PBT. Data represent the Division Index (i.e. the average number of cell divisions that a cell in the original population has undergone) of pre-activated CD8+ T cells +/- Standard Deviation. (Data are representative of three independent experiments. P-values ^*^ < 0.05). **(B)** Measure of PBT proliferation co-cultured with MDM2 macrophages targeting mAb treated polystyrene beads. MDM2 were challenged with polystyrene beads non coated as control or coated with either the anti-AMHRII FcKO or the anti-AMHRII 3C23K for 24 hours prior the co-culture for an additional 4 days with pre-activated cell trace violet loaded PBT. Data represents the Division Index (i.e. the average number of cell divisions that a cell in the original population has undergone) of pre-activated CD8+ T cells +/- Standard Deviation. (Data are representative of three independent experiments. P-values ^**^ < 0.01). “n.a” refers to non-activated and a. to activated T cells.

It is clearly established that macrophages within tumors suppress T cell anti-tumor activities. We made the hypothesis that the engagement of macrophages with 3C23K anti-AMHRII antibody alters their T cell suppressive function. To test this hypothesis, COV434-AMHRII target cells were treated with either the irrelevant mAb R565, the anti-AMHRII FcKO or the anti-AMHRII 3C23K mAb and co-cultured with MDM for 4 days prior addition of CD3/CD28 pre-activated PBT. CD8^+^ T cell proliferation was analyzed by the flow cytometry. As expected, in the presence of control mAbs (irrelevant isotype control R565 and FcKO anti-AMHRII mAbs) or in absence of treatment, MDM strongly impaired T cell proliferation. Of note, MDM mediated T cell immunosuppression was significantly reduced when co-cultured tumor cells were treated with 3C23K anti-AMHRII mAb as shown by the high increase of the division index of CD8 T cells (Figure 5A).

The decrease in tumor cell number as already shown in Figure 4A can partially explain this “immunostimulating” effect, as tumor cells are known to directly exert T cell suppressive functions. To test whether 3C23K anti-AMHRII mAb could also acts on MDM, rendering them less immunosuppressive we designed an experiment without tumor cells. Inert Sphero^®^ polystyrene beads were used as a substitute for tumor target cells. Those beads were treated with mAb in the same setting of tumors cells i.e. MDM were first co-cultured with mAbs treated beads prior co-culture with activated PBT. In these conditions, CD8^+^ T cell proliferation was partially restored when MDM were co-cultured with 3C23K coated Sphero^®^ polystyrene beads (Figure 5B). As a control, we checked that the T cell proliferation observed in the absence of MDM was not affected by 3C23K (Supplementary Figure 8). These experiments strongly suggest that 3C23K directly alters the T cell suppressive capacity of MDM.

Together, these results demonstrate that the humanized glyco-engineered monoclonal anti-AMHRII antibody, 3C23K, efficiently targets tumor cells by the antigen binding site and directs pro-tumor macrophages against tumor cells by the recognition of the Fc domain. Thus mAb activated macrophages trigger ADCC and ADCP against tumor cells and reduced their immunosuppressive behavior towards T cells.

### The 3C23K antibody displayed antitumor efficacy on ovarian cancer PDXs

3C23K was administered alone or in combination with the carboplatin-paclitaxel chemotherapy regimen. In all cases, we did not observe any toxicities in treated mice.

**Figure 6:**
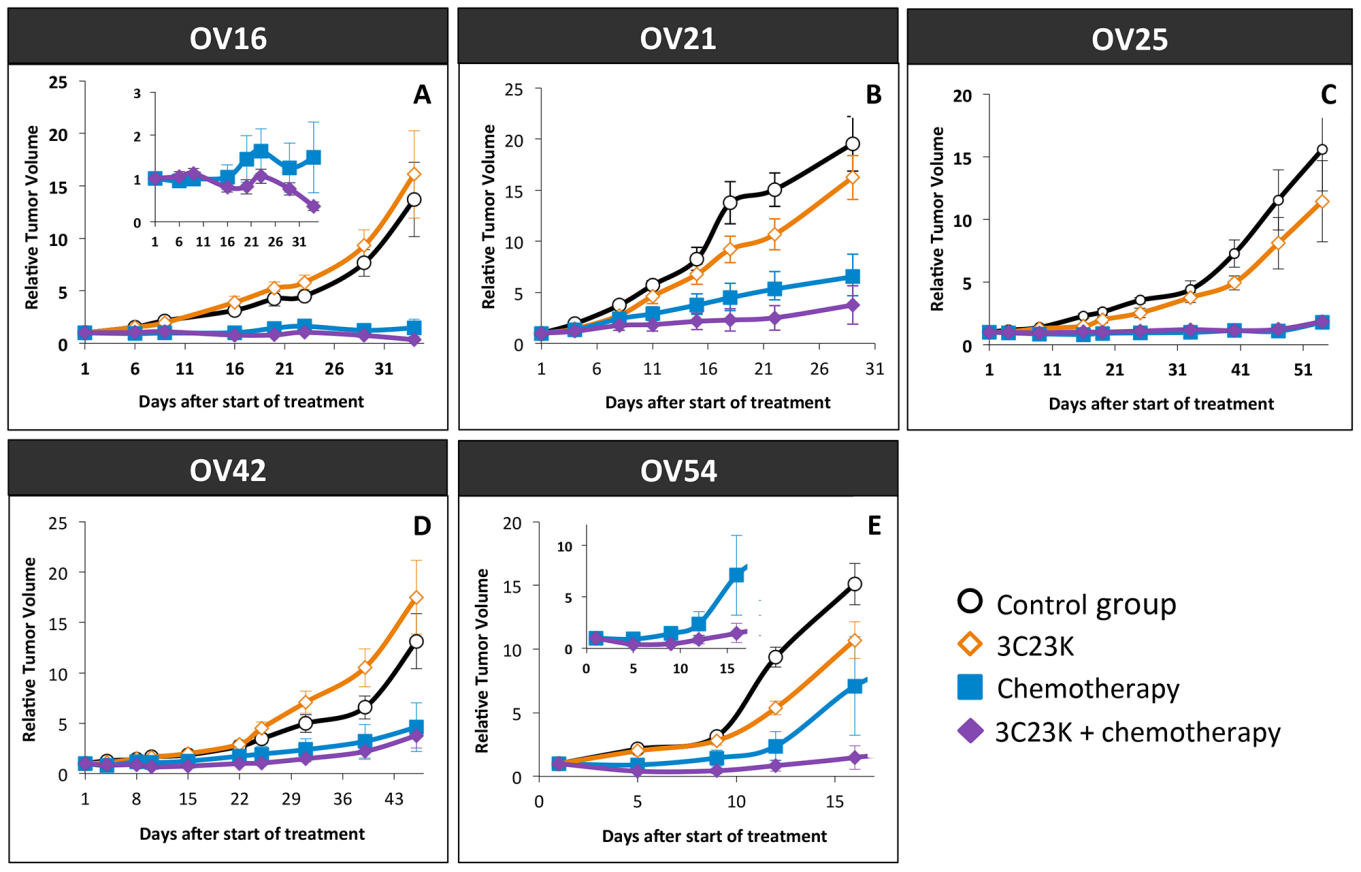
Tumor Growth Inhibition (TGI) after 3C23K administration in ovarian cancer PDXs Tumor growth was evaluated by plotting the mean of the RTV (relative tumor volume) ± SEM per group. Between 8 to11 mice per group were included in *in vivo* experiments. **(A)** Mice bearing OV16 xenografts were treated either by 3C23K at a dose of 20 mg/kg per injection, twice a week (◊), or by carboplatin at a dose of 66mg/kg and paclitaxel at a dose of 30 mg/kg per injection, every 3 weeks (■), or by carboplatin at a dose of 66mg/kg and paclitaxel at a dose of 66 mg/kg, every 3 weeks, combined with 3C23K at a dose of 20 mg/kg per injection, twice a week (♦). Mice in the control group (○) received 10 ml/kg of the 3C23K-formulating vehicle, PBS, with the same schedule as 3C23K treated animals. **(B)** Mice bearing OV21 xenografts were treated either by 3C23K (◊), or by carboplatin and paclitaxel (■), or by and paclitaxel at a dose, combined with 3C23K, (♦), as the same schedule as OV16. **(C)** Mice bearing OV25 xenografts were treated either by 3C23K (◊), or by carboplatin and paclitaxel (■), or by and paclitaxel at a dose, combined with 3C23K, (♦), as the same schedule as OV16. **(D)** Mice bearing OV42 xenografts were treated either by 3C23K (◊), or by carboplatin and paclitaxel (■), or by and paclitaxel at a dose, combined with 3C23K, (♦), as the same schedule as OV16. **(E)** Mice bearing OV54 xenografts were treated either by 3C23K (◊), or by carboplatin and paclitaxel (■), or by and paclitaxel at a dose, combined with 3C23K, (♦), as the same schedule as OV16.

**Figure 7:**
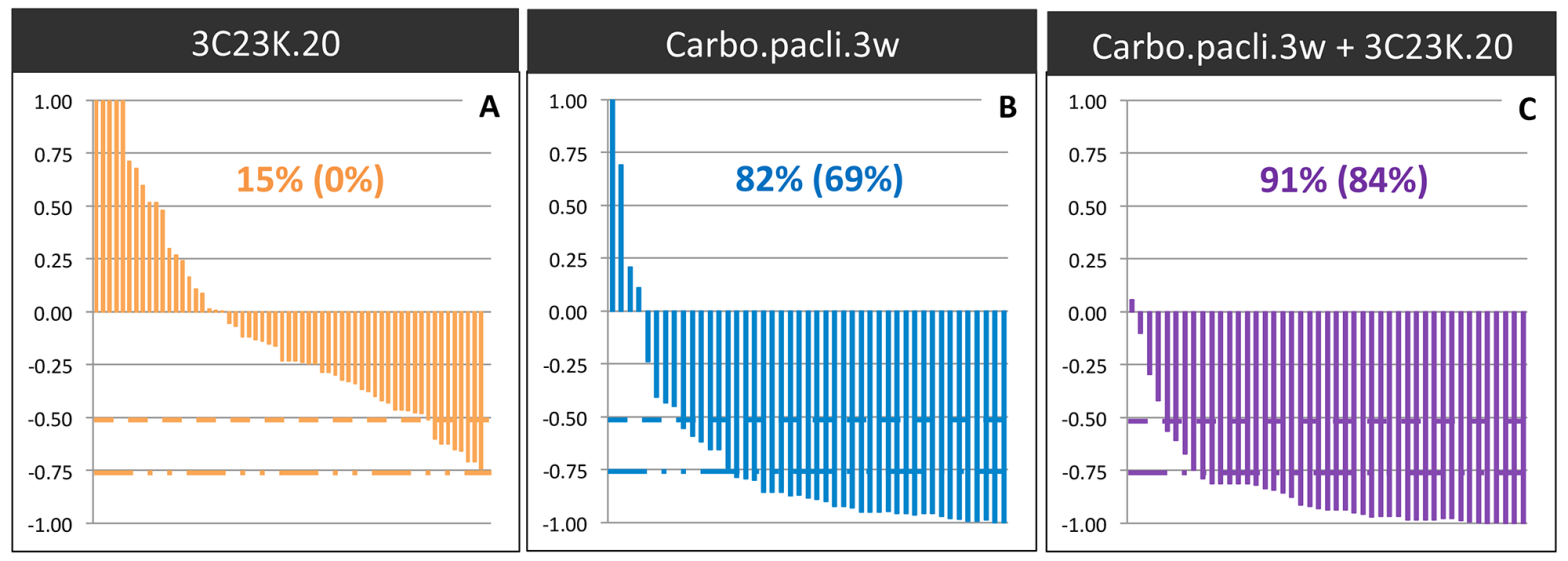
Overall Response Rate (ORR) after 3C23K administration in ovarian cancer PDXs The ORR was defined as the relative tumor volume variation (RTVV) of each treated mouse calculated from the following formula: [(Vt/Vc) –1], where Vt is the volume of the treated mouse and Vc the median volume of the corresponding control group at a time corresponding to the end of treatment.**(A)** ORR of mice treated by 3C23K at a dose of 20 mg/kg per injection, twice a week. **(B)** ORR of mice treated by carboplatin at a dose of 66mg/kg and paclitaxel at a dose of 30 mg/kg per injection, every 3 weeks. **(C)** ORR of mice treated by carboplatin at a dose of 66mg/kg and paclitaxel at a dose of 66 mg/kg, every 3 weeks, combined with 3C23K at a dose of 20 mg/kg per injection, twice a week. The two indicated percentages indicate the proportion of mice with a [(Vt/Vc) –1] lower than -0.5 and 0.75, respectively.

We first analyzed the antitumor effect of 3C23K administered alone at a dose of 20 mg/kg twice a week, for 2 to 7 weeks, in five different ovarian cancer PDXs (OV16, OV21, OV25, OV42, and OV54). The Tumor Growth Inhibition (TGI) ranged between 0% and 35% (Figure 6), with an ORR considered as significant in 15% (ORR < -0.5) (Figure 7). Moreover, we have evaluated the dose-dependent efficacy of 3C23K in one model, the OV54 PDX which was treated by 10 or 20 mg/kg per injection of antibody, and observed a trend but no significant difference of TGI, i.e. 8% and 25%, respectively (data not shown).

In a second step, we evaluated the efficacy of 3C23K when concomitantly administered with the standard chemotherapy carboplatin + paclitaxel. Chemotherapy was efficient in the five treated models, with TGI ranged between 80% and 95% and an ORR lower than -0.5 of 82% (Figure 6 and 7). In two models (OV21 and OV54), 3C23K at a dose of 20 mg/kg per injection, significantly increased TGI in comparison to chemotherapy alone, i.e. 89% *versus* 80% and 98% *versus* 86% in the OV21 and OV54, respectively (Supplementary Table 3) (Figure 6B and 6E). Moreover, in the OV16 and OV54 PDXs, the concomitant administration of 3C23K with chemotherapy significantly increased the proportion of CR, 67% and 60%, in comparison to chemotherapy alone, 37% and 10%, respectively (Supplementary Table 3). When considering all treated mice into the five PDXs, the proportion of CR was 13% after chemotherapy alone and 38% after 3C23K + chemotherapy (p < 10^-2^, χ2 test). Finally, the ORR lower than -0.5 was higher but not significantly after chemotherapy + 3C23K than chemotherapy alone (91% *versus* 82%) (Figure 7). It is remarkable that 3C23K also increased the quality of response to chemotherapy, with an ORR lower than -0.75 of 84% *versus* 69% (p < 0.1).

AMHRII expression was evaluated by cytometric analyses in 3 models (OV16, OV54, and OV5 [which was also previously treated with chemotherapy]) after 3C23K treatment, at time sacrifice (tumor size ∼ 2500 mm^3^). In all three cases, we did not observe modifications of tumor cell AMHRII expression after 3C23K (Figure 2B); in contrast, we observed a slight increase of AMHRII expression after chemotherapy (carboplatin + paclitaxel), and 3C23K (10 or 20 mg/kg per injection) + chemotherapy (Figure 2C). Similarly, we have also studied the stroma cell component after various *in vivo* treatments in the OV54 PDX, and did not observe modifications of macrophage orientation (M1 [CD11c and CD206] or M2 [CMHII]) (Figure 2D and 2E).

## DISCUSSION

In this study, we have assessed the mechanism of action and the efficacy of a new therapeutic approach in human epithelial ovarian cancers through AMHRII targeting. A first part of our study has been focused on the study of AMHRII expression, and a second part has been dedicated to its therapeutic efficacy tested in both ovarian cancer PDXs and in immune cell cultured assays (see Supplementary Figure 9 for a model).

As the treatment requires a direct interaction between AMHRII and 3C23K antibody, the expression of the receptor and its determination constitute one crucial step of clinical application. This issue has largely been investigated in our study, using three different methodologies, i.e. immunofluorescence on non-fixed fresh samples, flow cytometry and immunohistochemical analyses. The first two methods are totally dedicated to cell membrane expression since cells are not fixed nor permeabilized, while IHC allows cell membrane and cytoplasmic expression studies. Overall, we have shown that a large amount of human ovarian cancers express membranous AMHRII, 72% after IF determination and 96% after IHC study when considering a score of a least 100. These results are clearly concordant with previously reported studies where positive AMHRII expression was found between 69% and 93% of epithelial ovarian cancers [8, 9, 14, 15]. Further studies will be needed to investigate which threshold should be defined to discriminate positive and negative AMHRII tumors. As granulosa cancer cell tumors are considered as positive control for AMHRII expression [14, 16], the three specimens that we have studied by IHC showed a membranous score ranged between 100 and 240, suggesting that positive tumors could be formally defined by a threshold greater than 100. Hence, our study clearly confirms that human ovarian cancers expressed AMHRII.

To investigate this potential, we have studied the efficacy of 3C23K alone and in combination with the standard chemotherapeutic combination of carboplatin + paclitaxel in five ovarian cancer PDXs. While 3C23K induced a slight tumor growth inhibition when administered alone, it significantly increased chemotherapy-induced efficacy with an ORR lower than -0.5 of 91% *versus* 82% after chemotherapy alone; in particular, 3C23K increased quality of response with an ORR lower than -0.75 of 84% *versus* 69%, and 29% of complete remission *versus* 11% after chemotherapy alone (p < 0.05). Various studies have shown that AMH (also named MIS) induced *in vitro* [7, 14, 17-19] and *in vivo* [14, 15, 17, 18] antitumor activity. In particular, Chang et al. have demonstrated that AMH decreased *in vitro* invasion and *in vivo* migration of ovarian cancer cells [17]; similarly, Pieretti-Vanmarcke et al. showed that AMH increased efficacy of both *in vitro* and *in vivo* paclitaxel and cisplatin activity [18]. All these studies, based on physiological properties of AMH, have validated AMHRII as a target for treating ovarian cancers. Recently, strategies involving 12G4, a murine anti-AMHRII monoclonal antibody [14] and 3C23K, its human counterpart [10], showed antitumor activity against an AMHRII transfected GCT cell line, when used alone or in combination with carboplatin. Our preclinical results are in concordance with these previous studies. One can notice that *in vivo* experiments have been performed with immuno-deficient *Nude* mice that did not favor activation of immune system. Moreover, we showed that 3C23K interact with murine effector cells mainly *via* mFcγRIV. Particularly, 3C23K displayed a binding profile to Fcγ receptors similar to that described with other low-fucose IgG1, in accordance with that previously determined [10], with a 10 to 100-fold inferior affinity of 3C23K for most murine homologs of Fcγ receptors to those observed with the human receptors, as previously described for human IgG1 [20]. Of note, mFcγRIV is not expressed by mouse NK cells [21, 22], suggesting that 3C23K activity could involve in mice other immune effectors cells such as macrophages and neutrophils [20, 21, 23, 24]. Our data is in accordance with those demonstrating under 3C23K stimulation, an extremely reduced activation of murine NK, partially compensated by phagocytosis from murine macrophages [10]. Overall, all these data strongly suggest that *in vivo* experiments under-estimate efficacy of 3C23K that could be expected in a human context.

In addition, it could be possible that PDXs AMHRII expression artefactually decreased in *in vivo* models [15, 25]. Our IF and cytometry data also revealed that AMHR2 is expressed by a fraction of tumor cells, especially those that are positive for E-cadherin and CD44, a cancer stem cell marker, in line with previous studies [26-28]. Thus, the expression of AMHR2 by cancer cells known to be resistant to standard chemotherapy provides a scientific rationale for combination therapy with both 3C23K and chemotherapeutic agents in the treatment of advanced ovarian carcinoma. The possibility that AMHRII positive tumor cells correspond to tumor stem cells resistant to chemotherapy is an important point that would deserve further investigations.

Antitumor mAbs use a wide range of mechanisms to induce tumor elimination. Apart from direct antitumor effects, therapeutic mAbs can also exhibit indirect effects through the involvement of immune cells. It is particularly true with 3C23K, produced by EMAbling^®^ and displaying a fucosylation profile known to favour effector recruitment via an increased binding to human CD16a receptor [10]. In general, NK cells are considered as main effector cells for killing tumor cells via an ADCC process [29]. This mechanism might apply for haematological malignancies with mAbs bridging NK cells with tumor cells in the blood. However, the situation in solid tumors is probably different and our data suggest that in ovarian carcinomas, 3C23K engage macrophages in a Fc receptor-dependent mechanism resulting in the elimination of tumor cells. First, NK cells are scarce in solid tumors unlike monocytes and macrophages which are one of the most abundant populations found in ovarian carcinomas and ascites. In addition, we found that macrophages with a M2 like phenotype are localized close to tumor cells. It is now clearly established that these cells favor the growth of the tumor by multiple mechanisms and are associated with a bad outcome in a number of malignancies including ovarian carcinomas [30, 31]. Our data obtained with cultured cells and cells purified from patient ascites demonstrate that macrophages in presence of 3C23K but not with the Fc KO version of this mAb are able to eliminate tumor cells expressing AMHR2. These findings make tumor-associated macrophages candidates as mediators of the antitumor effects of 3C23K in ovarian carcinomas. The notion that macrophages are important cells in eliciting the antitumor effects of mAbs is supported by several studies. For instance, it has recently been shown that liver macrophages (Küpfer cells) are key effector cells for eliminating target cells that are present in the circulation [32, 33]. Even if the participation of macrophages in mediating antitumor mAb efficacy is less established in solid tumors, at least one report is consistent with this notion [34]. Of interest, M2 macrophages which are particularly enriched in OC, were described to express high levels of CD16a, the Fc receptor required for ADCC [35]. The mechanism by which macrophages kill tumor cells during mAb therapy is still a matter of debate. Studies performed with intravital microscopy showed that antibody-dependent cell phagocytosis (ADCP) is the main mechanism of action mediated by liver macrophages [32]. Our *in vitro* and *ex vivo* data are also suggestive of ADCP mechanism, at least during the first hours, with uptake of tumor cells and the establishment of larges vacuoles referred to as phagosomes. Macrophages engaged with 3C23K might also induce tumor cell killing via ADCC at later time points. Although ADCC is predominantly attributed to NK cells, it was proposed that monocytes and macrophages may also be capable of ADCC. For instance, synapse formation between tumor cells and macrophages was observed in peritoneal lavages of mAb-treated mice [36], suggesting the occurrence of ADCC. As a perspective of this study, it might be of interest to evaluate the *in vivo* efficacy of 3C23K combined with chemotherapies in macrophage-depleted models.

However, ADCC/ADCP might not be the only mechanism induced by macrophages upon mAb treatment. Tumor-associated macrophages have been described to suppress T cell activation [37] and our data showing contacts between lymphocytes and macrophages (Figure 3F and 3G) support the idea of direct crosstalk between both cell types. By using *in vitro* assays, we found that the engagement of FcR by 3C23K decreases the immunosuppressive phenotype of macrophages. In such conditions, pre-activated T cells regain their proliferative capacity that was blocked in the absence of 3C23K. The notion that therapeutic mAbs can engage innate but also adaptive immune cells is consistent with previous studies. In mouse tumor models, it was demonstrated that treatment with anti-tumor antigens Ab induced a cellular immune response, involving T cells, which was required for long-term survival [38, 39]. However, induction of adaptive immune responses in cancer patients that have been treated with anti-tumor mAbs has not yet been extensively investigated.

The mechanism by which 3C23K changes the phenotype of macrophages relieving T cell suppression is not known at present. However, different hypothesis can be envisioned. The interaction of antibodies with Fc receptors expressed by macrophages has been shown to trigger several signaling cascades that regulate the function of these cells [37]. Our preliminary data show that macrophages activated via FcR with 3C23K produce several pro-inflammatory cytokines including IL-1β and IL-6 that have been described to exert beneficial effect on T cells [40]. Indirect effects are also possible. In particular, the death of tumor cells can lead to the release of several danger-associated molecular pattern molecules (DAMPs) such as calreticulin which in turn activates innate and adaptive immune cells [41]. The role of dendritic cells in mediating this immunogenic cell death has been well described. Evidence also suggests that calreticulin released during cell death activates macrophages which produce IL-6 and TNF-a susceptible to exert beneficial effects on T cells [42].

The fact that 3C23K antibody produce antitumor effect in combination with cytotoxic molecules raises the interesting hypothesis that the ADCC and ADCP functions of macrophages work better on damaged cells. A number of chemotherapeutic molecules such as those used in our study have been shown to induce immunogenic cell death which, as stated above, can change the phenotype of macrophages.

In conclusion, our study has clearly experimentally confirmed the potential interest of AMHRII targeting in human ovarian cancers. This conclusion is supported by our AMHRII expression analyses in human ovarian tumor samples, preclinical activity of the specific mAb 3C23K directed against AMHRII, and immunological impact of the antibody. A phase I/II clinical study has just been initiated to evaluate the potential of 3C23K in gynecologic cancers. From a clinical perspective, various issues are currently raised, i.e. efficacy of treatment combinations, immunological therapeutic potential of 3C23K, and new clinical indications over the well-known human gynecological cancers [19, 43-45], such as endometriosis [46, 47] or breast, prostate or lung cancers [48, 49].

## MATERIALS AND METHODS

### Patient’s ovarian cancer tumors

Various fresh ovarian tumors obtained from anonymized ovarian cancer patients have been included in the study, as detailed in Supplementary M&M.

### Ovarian cancer preclinical models

The human GCT cell line COV434-AMHRII clone that stably expresses high AMHRII levels was described by Kersual *et al.* [14]. *In vitro* culture protocol is described in Supplementary M&M.

For *ex vivo* experiments, 26 different ovarian cancer Patient-Derived Xenografts (PDXs) have been used. Five PDXs have been included in the *in vivo* experiments, namely the OV16, OV21, OV25, OV42, and OV54 models. The main features of these PDXs, described in the Supplementary Table 4, showed a strict histopathological correlation between PDXs and their corresponding patient’s tumors.

### Purification of Human immune cells

Purification of Human PBMC, T cells and monocytes as well as the generation of M2 type macrophages are described in Supplementary M&M.

### Tumor dissociation protocol of OC PDXs

Three different materials were used for cytometry: fresh patient tumors or OC PDX and patient ascites. To obtain isolated cells from fresh PDX or patient tumors, 2 protocols were used. Both of tumors were minced mechanically. For PDX, a protocol published elsewhere [50] were adapted to OC PDXs and detailed in Supplementary M&M.

### Cytofluometric analyses of fresh patient’s tumors

Cells from fresh patient’s tumors or from PDX were phenotyped by flow cytometry after staining with a combination of antibodies. For details see Supplementary M&M.

### Quantitative analysis of cellular AMHRII expression by flow cytometry

Quantitation of AMHRII binding sites was performed on tumor patient’s biopsies and available PDXs. Protocol is described in Supplementary M&M.

### *In vitro* and ex vivo immunological assay

ADCC/ADCP was evaluated using fluorescent imaging. *In vitro* assays consist of COV434-AMHRII target cells put into contact with MDM effector cells. *Ex vivo* assays consist of COV434-AMHRII target cells put into contact with total ascites cells from ovarian cancer patients. For details see Supplementary M&M.

T cell proliferation assay is described in Supplementary M&M.

### Immunofluorescence analyses

Tumor slices were prepared as previously described [51, 52]. This assay consists of thick slices (400 μm) made from unfixed human ovarian tumors, subsequently immunostained with a combination of antibodies. For details see Supplementary M&M.

### Surface plasmon resonnance (SPR) analysis

Binding profile of 3C23K to human and murine Fcγ receptors was determined by SPR and detailed in Supplementary M&M.

### Immunohistochemical analyses

Paraffin-embedded tissue blocks, obtained at the time of the initial diagnosis were retrieved from the archives of the Department of Biopathology of Curie Hospital. Immunohistochemical protocol is detailed in Supplementary M&M. AMHRII expression was studied at both membranous and cytoplasmic cellular levels. For each tumor sample, the proportion of positive cells and its corresponding immunostaining intensity was defined, allowing determination of a membranous and cytoplasmic scores that were defined by the following formula: % of AMHRII-positive tumor cells (%) x intensity of immunostaining (I). Hence, each score ranged between 0 and 300.

### *In vivo* experiments

The humanized anti-mullerian Antibody, 3C23K, was obtained from Gamamabs Pharma (France) from 12G4 a murine of which the pharmacological profile has been described previously [12]. 3C23K is a glyco-engineered antibody with a low-fucose content allowing a high affinity to CD16 receptor and consequently a high potential for immune cell engagement such as macrophages and NK cells [10]. *In vivo* protocols are detailed in Supplementary M&M.

### Statistical tests for *in vivo* experiments

All statistical tests are detailed in Supplementary M&M.

## SUPPLEMENTARY MATERIALS FIGURES AND TABLES


