# Supplementary material for: The humanized anti-human AMHRII mAb 3C23K exerts an anti-tumor activity against human ovarian cancer through tumor-associated macrophages
Source: Oncotarget. 2017 Oct 7;8(59):99950–65. doi: 10.18632/oncotarget.21556 (PMC5725143; doi:10.18632/oncotarget.21556)
Supplement: Supplementary file 1 [file oncotarget-08-99950-s001.pdf]

The humanized anti-human AMHRII mAb 3C23K exerts an anti-tumor activity against human ovarian cancer through tumor-associated macrophages

SUPPLEMENTARY MATERIALS

Patient's ovarian cancer tumors

Fresh ovarian tumors obtained from anonymized patients diagnosed with clinical stage I-IV epithelial ovarian cancer and who underwent exploratory laparotomy or complete surgery at the European Georges Pompidou Hospital, Paris. Non-fixed fresh tumors obtained after the resection were rapidly transported to the laboratory in ice-cold RPMI 1640 and analyzed within the next 24 hours. A total of 34 epithelial ovarian cancers were used for this study. Patient's characteristics are summarized in the supplementary table 2. Human tumors were obtained with the agreement of the ethical committee of the Assistance Publique-Hôpitaux de Paris (AP-HP), in application with the article L.1121-1 of French law. Ascites from 9 other OC patients were collected at the unit of Oncology at Cochin Hospital, Paris and at Gustave Roussy Hospital, Villejuif (written informed were obtained from all patients included). Finally, twenty-six other patient's ovarian cancer tumors originated from the Institut Curie have been included in the immunohistochemical determination of AMHRII expression.

Ovarian cancer preclinical models

Cells were grown in DMEM F12 medium containing 10% heat-inactivated fetal bovine serum, 0.1 mg/ml streptomycin, 0.1 IU/ml penicillin. For COV434-AMHRII cells, the medium was supplemented with 0.33 mg/ml Geneticin to maintain selection. Cells were grown at 37°C in a humidified atmosphere with 5% CO2 and medium was replaced twice a week. Cells were harvested with 0.5 mg/ml trypsin/0.2 mg/ml EDTA. All culture media and supplements were purchased from Life Technologies, Inc. (Gibco BRL, Gaithersburg, MD).

Purification of Human immune cells

Human PBMC were isolated from whole blood of healthy donors (n=18, collected at the Etablissement Français du Sang) by ficoll-gradient separation. Monocytes were isolated from PBMC by negative selection to prevent cell activation (CD14 without CD16 depletion STEMCELL Technologies Easy-Sep® Kit) and cultured 6 or 7 days in RPMI medium containing 10% heat-inactivated AB human serum, hepes 0.1 mM, 0.1 mg/ml streptomycin, 0.1 IU/ml penicillin, supplemented with 100 ng/ml of human M-CSF (Preprotech®) to allow M2 macrophage differentiation. Monocytes derived M2 type macrophages (MDM2) were obtained by differentiation under M-CSF during 7 days of culture. After 7 days of differentiation, these cells express M2 like markers such as CD206 and CD163 (Supplementary Figure 5).

Human T lymphocytes were isolated from whole blood of healthy donors (n=6, collected at EFS) by ficoll-gradient separation. T cells were isolated from PBMC by negative selection (Human T Cell Enrichment Kit STEMCELL Technologies Easy-Sep®) and cultured in RPMI medium containing 10% heat-inactivated AB human serum, hepes 0.1 mM, 0.1 mg/ml streptomycin, 0.1 IU/ml penicillin during 1 to 3 days before activation by T-activator CD3/CD28 Dynabeads (Gibco®, LifeTechnologies™).

Tumor dissociation protocol of OC PDXs

Briefly, tumors were immersed in non-enzymatic buffer (invitrogen 13150-016), cruched mechanically, incubated for 20 minutes at RT, twice. The digestion was completed by enzymes (Collagenase sigma c6885 + DNase invitrogen 18047-019) mechanically crushed. Finally tumor cells were purified on density gradient (Ficoll histopaque sigma 11191). All PDX except the mucinous one, OV21, were dissociated with this method. For Patients biopsies, enzymatic dissociation was done with 62.5 µg/ml Liberase® (Roche) and 100 µg/ml DNAse I (Roche) at 37°C in RPMI 1640 for 45 minutes. Finally, ascites were centrifuge and freshly used for further immunostaining. Red cells were lysed by 2 min treatment with ACK lysis Buffer (150 mM AmmoniumChlorid (NH4Cl), 10 mM Kaliumhydrogencarbonat (KHCO3), 1mM EDTA-2Na).

Cytofluometric analyses of fresh patient's tumors

Total cells were phenotyped by flow cytometry after staining with the following antibodies: AlexaFluor488-conjugated 3C23K or the isotypic control AlexaFluor488-conjugated R565 (conjugated by GlycoDiag, France

for GamaMabs Pharma), Anti-EpCam BV421, CD45 APC-Cy7, CD15 PerCP, CD16 APC-Cy7 (Biolegend®), anti-CD11b PE-Cy7, CD3 PerCP, CD8 PE, CD4 Pacific Blue, CD56 PE, CD20 Fitc (BD Pharmingen®), CD14 Fitc, HLA-DR Pacific Blue (MACS Miltenyi®), CD206 PE (eBoscience®). Dead cells were excluded by Fixable Viability Dye eFluor® 506 (eBioscience®) staining prior antibodies stainings. Samples acquisition was performed on a FACS Canto II cytometer (BD) and data were analyzed on FlowJo (TreeStar, version 10.0.7).

Cytometry analyses of PDXs were performed for AMHRII staining and mouse stromal components. Hence, the AMHRII staining protocol included 3C23K/R565 antibodies purified or conjugated to AF488 (invitrogen A11013). In order to segregate by analyses human cells from mouse ones, a multicolor gating was realized, using hEpCAM_perCP-Cy5.5 (Biolegend 324213), mCD45_PE-TR (eBioscience 61-0459), and DAPI (ThermoFischer D1306) (Supplementary Fig. 10A). To evaluate the proportion of immune stromal cells within our ovarian cancer PDXs, a set of antibodies was used to point out main components, such as B-cell lymphocytes (CD19_PE; Biolegend 115519), monocytes (LY6C_APC-H7; BD Pharmingen 560596), macrophages (F4/80_APC; eBioscience 17-4801-82), granulocytes (LY6G_FITC; BD Pharmingen 551460), and NK lymphocytes (CD49b_PE-Cy7 (DX5); Biolegend 108907). To distinguish M1 like (phenotypically inflammatory) from M2 like Tumor associated macrophages (TAMs), we have also included 3 other antibodies, i.e. CD11c_PE-Cy7 (eBioscience 25-0114-81) and CD206_FITC (Biolegend 141710) for M1 like-macrophages, and CMHII_APC-H7 (Biolegend 107627) for M2 like-macrophages. Cytometry analyses were performed on a BD LSRII cytometer. Retained unit for the expression of receptor was the specific Mean Fluorescent Intensity (MFI: arbitrary unit of signal to noise ratio from measured fluorescence) stated as the Geometric Mean (adapted for logarithmic distributions) of majority human population stained with 3C23K weighted with MFI of this population stained with R565 (non-specific signal).

Quantitative analysis of cellular AMHRII expression by Flow cytometry

The quantitation of AMHRII binding sites on resuspended tumor cells was performed using The QuantumTM Simply Cellular (Bangs Laboratory) according to the manufacturer's instructions. Briefly, the four microbeads populations labeled with a different calibrated amount of mouse anti-human IgG specific for the Fc portion of human IgG antibodies were stained with the same AlexaFluor488-conjugated anti-AMHRII 3C23K and under the same conditions as the cell samples. Each bead population binds varying amounts of the AlexaFluor488-conjugated anti-AMHRII 3C23K, producing a corresponding intensity of fluorescence, which is analyzed on a FACS Canto II cytometer (BD). A calibration curve was generated by plotting the mean fluorescence intensity of each bead population versus its assigned Antibody Binding Capacity (ABC). Resuspended tumor cells are then analyzed on the flow cytometer using the same instrument settings as for the calibration beads and the ABCs of the cell populations are calculated by interpolation from the calibration curve. Quantification of the AMHRII receptor in PDXs was performed using beads kit QuantumTM Simply Cellular anti-human IgG (BangsLaboratories cat.816).

In vitro and *ex vivo* immunological assay

For *in vitro* ADCC/ADCP, COV434-AMHRII target cells were loaded with a green fluorescent dye (i.e. CMFDA, Molecular Probes®, Life Technologies™) and MDM effector cells were loaded with an U.V. fluorescent dye (i.e. Cell Trace Violet, Molecular Probes®, Life Technologies™). Target (T) and effector (E) cells were co-cultured at various E/T ratios with various concentration of either the irrelevant mAb R565 (GamaMabs Pharma, France) or the anti-AMHRII Fc-mutated counterpart of 3C23K mAb (named FcKO; GamaMabs Pharma), or the anti-AMHRII 3C23K mAb itself GamaMabs Pharma). Cells were plated on µ-slide chambers (Ibidi®) to allow direct and real-time visualization and quantification of cell number by IF.

For *ex vivo* ADCC/ADCP assay, total ascites cells were collected after centrifugation and resuspended in RPMI medium containing 10% heat-inactivated AB human serum, hepes 0.1 mM, 0.1 mg/ml streptomycin, 0.1 IU/ml penicillin. Then ascites cells were plated on 96-well plate and co-cultured 6 days with CMFDA stained COV434-AMHRII target cells in presence of either the irrelevant mAb R565 or the anti-AMHRII FcKO, or the anti-AMHRII 3C23K mAb. Samples acquisition was performed on a FACS Canto II cytometer (BD) after anti-CD14 APC-Cy7, CD206 PE, and CD163 APC Ab stainings. Dead cells were excluded by Fixable Viability Dye eFluor® 506 (eBioscience®) staining prior antibodies stainings. Tumor cell numbers were determined as the simple CMFDA positive cells. Net phagocytosis was determined as the percentage of double CMDA/CD14 positive cells among total CMFDA positive cells. TAMs were phenotyped by CD14, CD206, and CD163 expression.

T cell proliferation assay was performed as follows. Briefly, CMFDA stained COV434-AMHRII were treated 1h at 4°C with 10 µg/ml of either the irrelevant mAb R565

or the anti-AMHRII FcKO, or the anti-AMHRII 3C23K mAb and incubated with unstained MDM2 for 4 days prior addition of CellTrace Violet (Molecular Probes®, Life Technologies™) stained T cells pre-activated by CD3/CD28 Dynabeads at MDM2: T cell ratio of 1:8. After 4 days of additional incubation period, cells were harvested and stained with anti-CD8 PerCP, CD11b PE-Cy7, and CD4 AF647 (BD Pharmingen®) before flow cytometry analysis. Dead cells were excluded by Fixable Viability Dye eFluor® 506 (eBioscience®) staining prior antibodies stainings. T cell proliferation was analyzed on CD8+ (CD11b-) T gated cells by the measure of CellTrace Violet dilution corresponding to cells divisions. The Division Index equivalent to the average number of cell divisions that a cell in the original population has undergone was calculated with FlowJo (TreeStar, version 7.6.5). The Division Index equivalent to the average number of cell divisions that a cell in the original population has undergone was calculated.

Immunofluorescence analyses

For preparation of tumor slices, fresh non-fixed samples were embedded in 5% low-gelling-temperature agarose (type VII-A, Sigma-Aldrich) prepared in PBS. 400-µm slices were cut with a vibratome (VT 1000S, Leica) in a bath of ice-cold PBS. Slices were transferred to 0.4-µm organotypic culture inserts (Millicell, Millipore) in 35-mm Petri dishes containing 1ml RPMI 1640 in an incubator at 37°C/5% CO2.

Live vibratome sections were stained for 15 minutes at 37°C with the following antibodies: AlexaFluor488-conjugated anti-AMHRII 3C23K or AlexaFluor488-conjugated R565, BrilliantViolet421-conjugated anti-EpCAM (clone 9C4, purchased from BioLegend), AlexaFluor647-conjugated anti-CD16 (clone 3G8, BD Pharmingen), Phycoerythin-conjugated anti-CD206 (clone 19.2, eBioscience), BrilliantViolet510-conjugated anti-CD90 (clone 5E10), PerCP/Cy5.5-conjugated anti-CD15 (clone W6D3, BioLegend), AlexaFluor488-conjugated anti-CD3 (clone UCHT1, BD Pharmingen) and DAPI (Sigma) and washed thereafter. All antibodies were diluted in RPMI and used at a concentration of 10 µg/ml.

Acquisitions of IF images were performed with a Spinning Disk inverted confocal microscope (LEICA) equipped with a Yokogawa CSU-X1M1 head, 4 excitation lasers (405, 491, 561, and 633-nm), and a CoolSnap HQ2 camera. Two oil immersion objectives were used: 20X, HCX PL APO C, numeric aperture: 0.7 and 63X, PLAN APO, numeric aperture: 1.4. Acquisition software: MetaMorph 7 (Molecular Devices). Analysis software: ImageJ 1.46n (Wayne Rasband, NIH, USA). An IF semi-quantitative score, ranging from - to +++, was attributed according to the AMHRII intensity of fluorescence with a defined cell surface expression for each sample. This scoring did not take into account the expression of the tumor marker EpCAM. It only concerns the cells with a membrane form of AMHRII since intracellular AMHRII was detected on non-fixed vibratome sections.

Surface Plasmon Resonance (SPR) analysis

Anti-histidine antibodies (R&D Systems) were immobilized on a T200 apparatus at 25°C in HBS-EP at 10µl/min flow rate on a CM5 sensor chip using EDC/NHS activation, according to the manufacturer's instructions (GE Healthcare). They were covalently immobilized at the 6900RU level on the flowcell Fc2 and a control reference surface (flowcell Fc1) was prepared using the same chemical treatment but without anti-His antibodies.

All kinetic measurements in Fc1 and Fc2 were performed by single-cycle titration on a T200 apparatus at 25°C in HBS-EP at 100µl/min. Each human and murine gamma receptor (R&D Systems) was captured on immobilized anti-His antibodies at 20nM during 60s. Five increasing concentrations of antibody were injected (injection time = 120s). After a dissociation step of 600s in running buffer, sensor surfaces were regenerated using 5µl of glycine-HCl pH1.7. All the sensorgrams were corrected by subtracting the low signal from the control reference surface and buffer blank injections. Kinetic parameters were evaluated from the sensorgrams using a heterogeneous ligand or two states models from the T200 evaluation software.

Immunohistochemical analyses

Immunohistochemical experiments were performed as follow: sections of 3 µm in thickness were cut with a microtome from the paraffin-embedded tissue blocks. Tissue sections were dewaxed and rehydrated through a series of xylene and ethanol washes. Briefly, primary antibodies (abs) against 3C23K biotinylated were used. Immunostaining was processed by using a Dako automated. The specificity of the AMHR2 abs was confirmed via the same protocol on paraffin-embedded human tissue sections and cells blocks sections. Anti-AMHR2, pH9, at 7µg/ml immunohistochemical analysis: After hydration in PBS solution for 5 minutes, application of the anti-AMHR2 for 1 hour incubation, revelation using a complex AB coupled to a peroxidase labeled (Vector laboratories), revelation of peroxidase after application of a DAB solution (Dako K3468) for 5 minutes, and Hematoxylin Mayer for 1 minute and then in mounting resin. Routinely fixed, paraffin-embedded, 3 µm-thick tissue sections were de-paraffinized, rehydrated and then unmasked in target retrieval solution at pH6 (EpCAM)

or pH9 (CD45) (15' at 95°C). Anti-EpCAM clone 1B7 (Thermofisher) and anti-CD45 clone UCHL1 (Agilent) detection by immunoperoxidase technique and DAB chromogenic substrate revelation on the BOND RX using the Bond Polymer Refine Detection Kit (Leica) according to the protocols recomended by the manufacturer: After blocking endogenous peroxydase activity and inhibiting non-specific staining, the slides were incubated with diluted antibody (1/200) for 15 minutes at room temperature. The tissue sections were then washed with PBS and incubated with polymeric horseradish peroxidase (HRP)-linker antibody conjugate system for 8 minutes. Immunoreactive signals were detected using 3,3'-Diaminobenzidine tetrahydrochloride hydrate (DAB) substrate solution. The substrate chromogen, DAB, visualizes the complex via a brown precipitate. Finally, the sections were lightly counterstained with Mayer's Hematoxylin.

In vivo experiments

3C23K was diluted in saline phosphate buffer (PBS). It was administered intraperitoneally, twice a week, for 6 to 19 weeks, depending on models and experiments. The control groups in all experiments were treated with PBS (vehicle) with the same schedule of administration as 3C23K. Carboplatin (Fresenius Kabi, France) was administered intraperitoneally at a dose of 33 or 66 mg/kg, at days 1 and 22. Paclitaxel (Sandoz, France) was administered intraperitoneally at a dose of 15 or 30 mg/kg, at days 1 and 22, or at a dose of 12 mg/kg, weekly, for 6 weeks. Doxorubicin (Teva, France) was administered intraperitoneally at a dose of 2 mg/kg, at days 1 and 22.

In vivo toxicity was assessed, as follow: Weights of individual mice will be measured daily for the first week, then twice a week. Variations of weight of mice as compared to their initial weight and means (or median) per group will be calculated. Treatments will be stopped once a bodyweight loss of 15% is observed, and animals will be sacrificed if bodyweight loss persists for three consecutive days. In contrast, animals will immediately be sacrificed in case of a bodyweight loss greater than 20%. A treatment will be considered as toxic in case of at least one death or in case of a weight loss greater than 15% of the initial weight.

Four to six week-old Nude mice, bred at Institut Curie, were used. Tumor fragments of 30-50 mm^3 were grafted subcutaneously into the interscapular fat pad. When tumors reached a size of about 50-100 mm^3, mice were randomly assigned to control or treatment groups. Between 8 and 10 mice per group were included in each experiment. Mice were sacrificed when tumor reached a volume of 2500 mm^3.

Tumor growth was evaluated by measuring with a caliper two perpendicular tumor diameters twice a week. Individual tumor volume, relative tumor volume (RTV) and tumor growth inhibition (TGI) were calculated according to a standard method [1]. To evaluate the response to each treatment according to individual mouse variability, we have considered each mouse as one tumor-bearing entity. We have defined a relative tumor volume variation (RTVV) of each treated mouse: RTVV = RTVt/RTVc, where RTVt is the RTV of the treated mouse and RTVc the median RTV of the corresponding control group at the day corresponding to the first ethical mice sacrifice. For each mouse, we calculated an overall response rate (ORR) using the formula: ORR= [(RTVV)-1]. A tumor was considered as responding to therapy when ORR was lower than -0.5. Since data were normalized to each control group, results of independent *in vivo* experiments could be merged. Survival curve was obtained with a cut off volume defined for each experiment. Median survival was obtained when 50% of tumor reached this volume.

Studies have been performed in compliance with protocol and animal housing in accordance with national regulation and international guidelines and under the supervision of authorized investigators. The experimental protocol and animal housing were in accordance with institutional guidelines as put forth by the French Ethical Committee (Agreement C75-05 - 18, France).

Statistical tests for *in vivo* experiments

Two by two comparison of TGI was done using a two-tailed Mann-Whitney test based on the RTVV. For all pairwise comparisons based on the proportions of tumors with a particular RTV or ORR, a two-tailed Fisher's exact test was used. All statistical tests were realized bilaterally calculating two-tailed p-values. Results were considered statistically significant when $p \leq 0.05$ (95% confidence interval). Wilcoxon and Log Rank was used for survival curves.

REFERENCES

1. Némati F, Sastre-Garau X, Laurent C, Couturier J, Mariani P, Desjardins L, Piperno-Neumann S, Lantz O, Asselain B, Plancher C, Robert D, Péguillet I, Donnadieu MH, Dahmani A, et al. Establishment and characterization of a panel of human uveal melanoma xenografts derived from primary and/or metastatic tumors. Clin Cancer Res. 2010 Apr 15;16:2352-62. https://doi.org/10.1158/1078-0432.CCR-09-3066. Erratum in: Clin Cancer Res. 2010 Jul 15;16:3807.

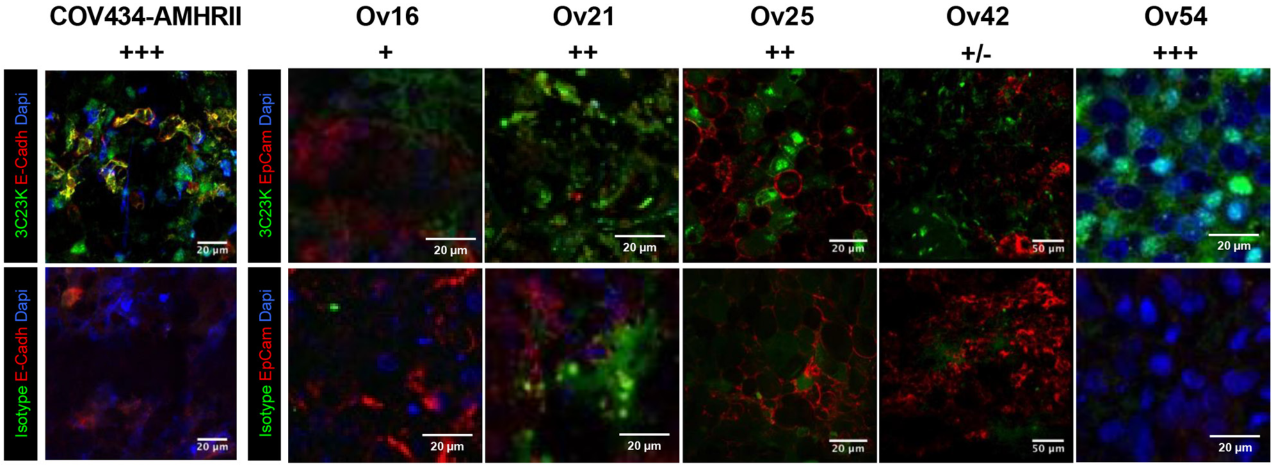

Supplementary Figure 1: Detection of AMHRII expression in PDX human ovarian tumors by the 3C23K mAb by IF. Representative multiplexed immunofluorescence microphotographs showing AMHRII detection by the AlexaFluor488 conjugated 3C23K mAb (green fluorescence channel, upper panels) compared to the AlexaFluor488 conjugated R565 isotype control mAb (green fluorescence channel, lower panels) in the COV434-AMHRII transplanted model and the 5 PDX ovarian tumor models, ranging from negative to high AMHRII expressing tumors. A qualitative score (+/- to +++) was attributed for each sample. Tumor nests were identified by EpCAM expression (red fluorescence channel). Nuclei were stained with DAPI (blue fluorescence channel).

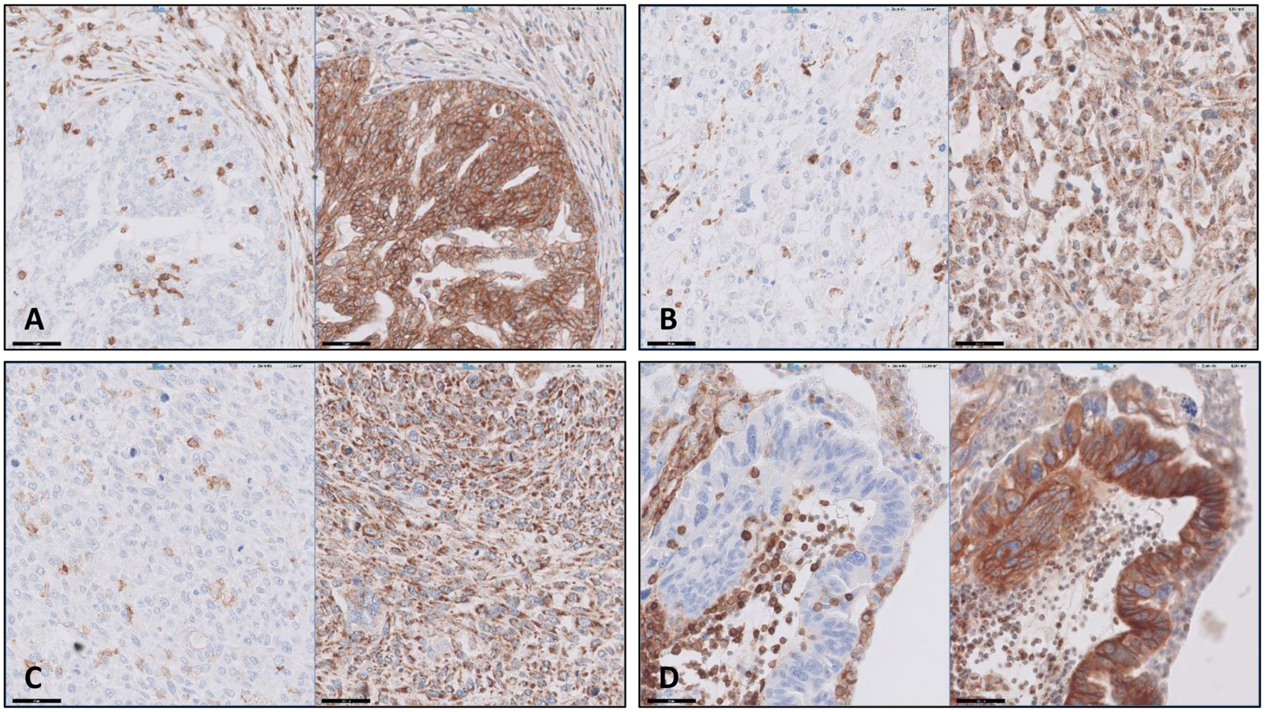

Supplementary Figure 2: Immunohistochemical determination of EpCAM and CD45 expression at the surface of PDX human ovarian tumor models. OV8 **(A)**, OV42 **(B)**, OV54 **(C)** and OV57 **(D)** PDX human tumors were immunostained with anti anti-CD45 antibodies (left panels) or anti-EpCAM antibodies (right panels). Immunsotaining was processed by using a Dako automated.

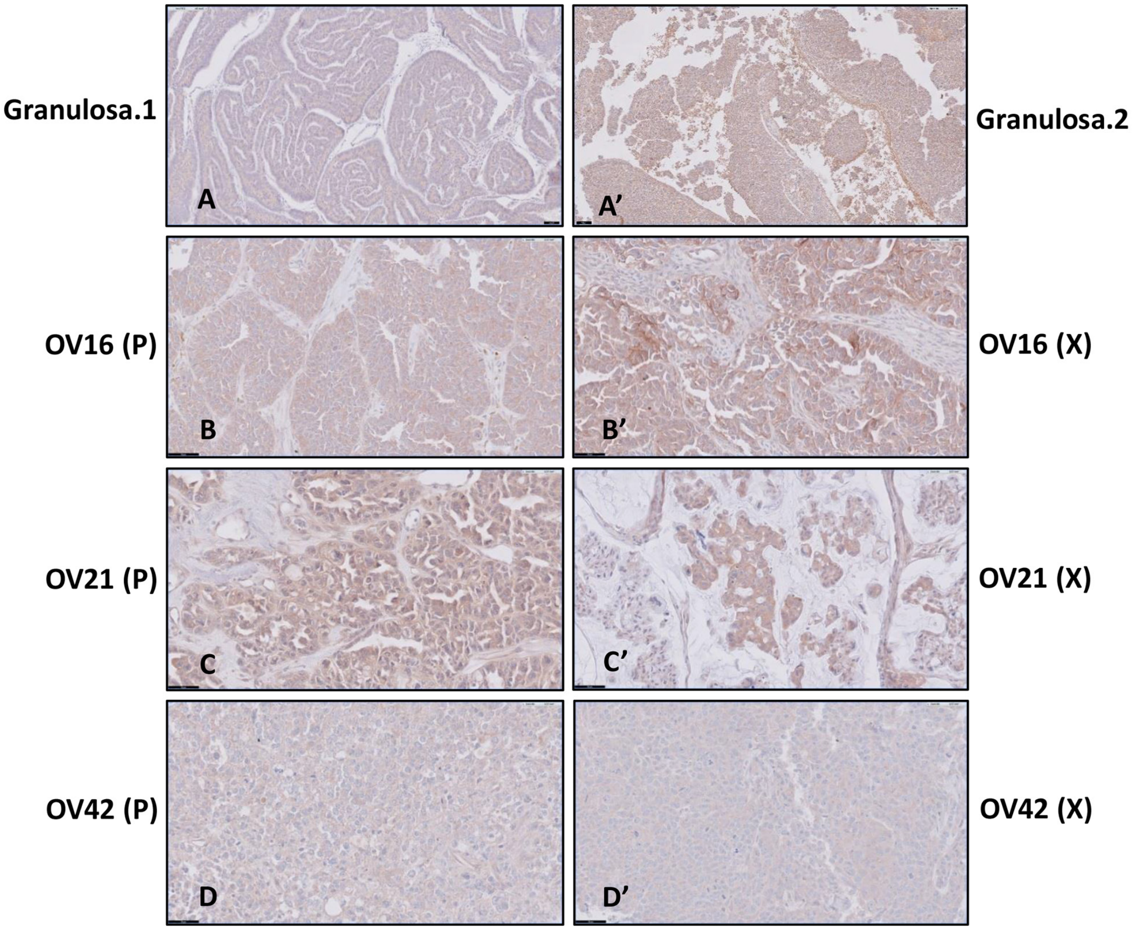

Supplementary Figure 3: Immunohistochemical determination of AMHRII expression. **(A)(A’)** Granulosa tumors. **(B) (B’)** Ovarian cancer OV16-patient tumor and its corresponding PDX. **(C) (C’)** Ovarian cancer OV21-patient tumor and its corresponding PDX. **(D) (D’)** Ovarian cancer OV42-patient tumor and its corresponding PDX. Primary antibodies (abs) against 3C23K biotinylated were used and immunostaining was processed by using a Dako automated.

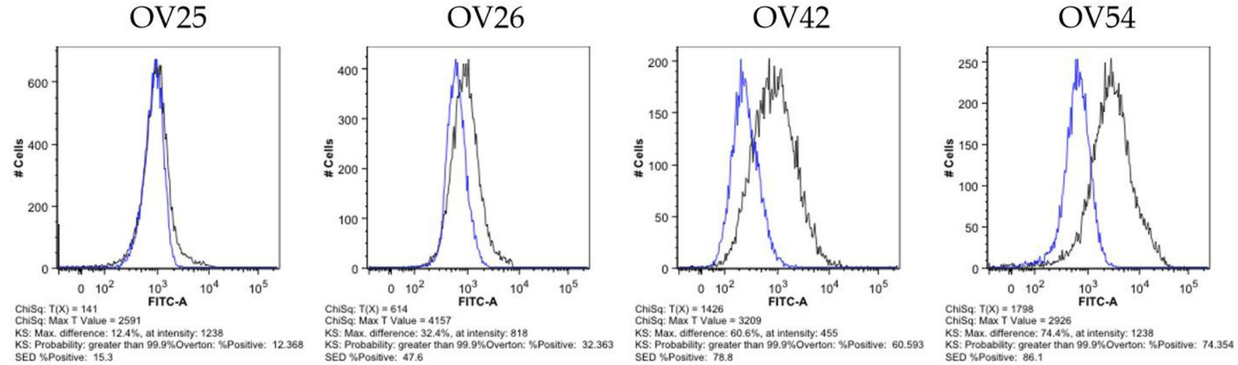

Supplementary Figure 4: AMHRII expression determined by flow cytometry at the surface of PDX tumor cells OV25, OV26, OV42 and OV54.

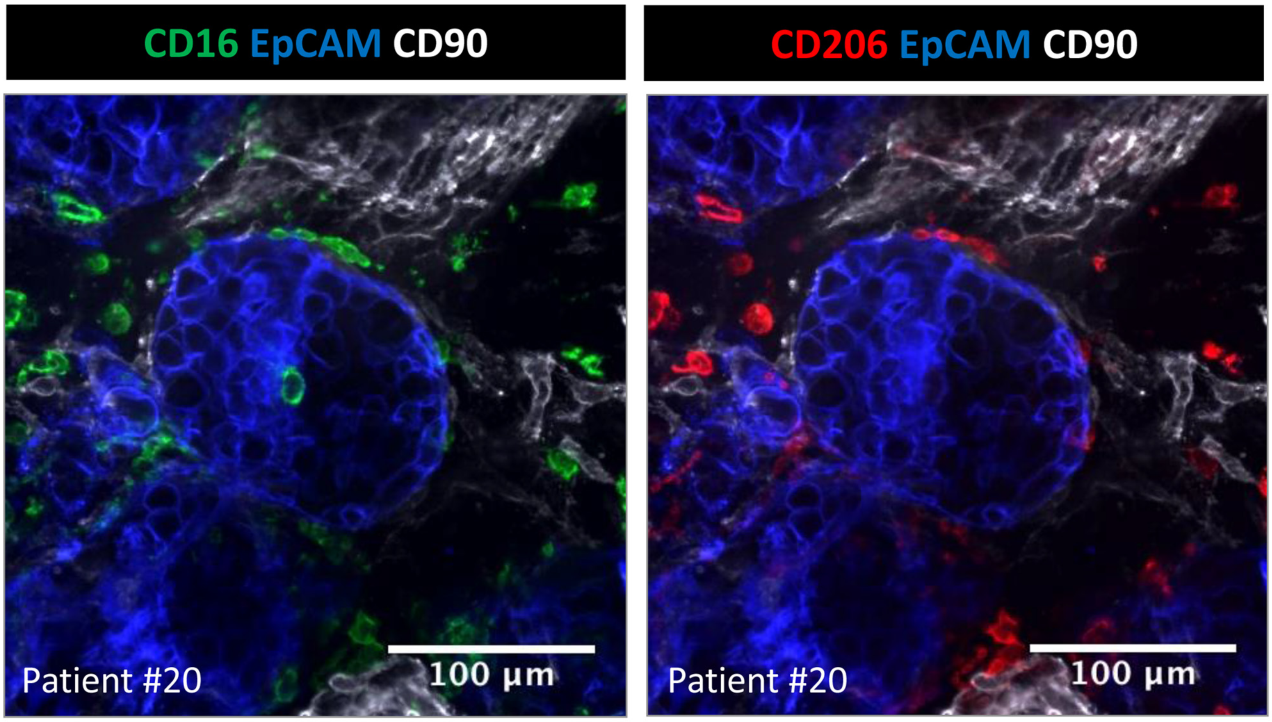

Supplementary Figure 5: Characterization of tumor-associated macrophages by immunofluorescence. Non-fixed vibratome sections of a human carcinoma (patient #20) were immunostained with the indicated antibodies. Note that most of the CD16+ cells in the tumor stroma also express CD206. CD90 also known as Thy1 is a marker expressed by stromal fibroblasts and activated endothelial cells.

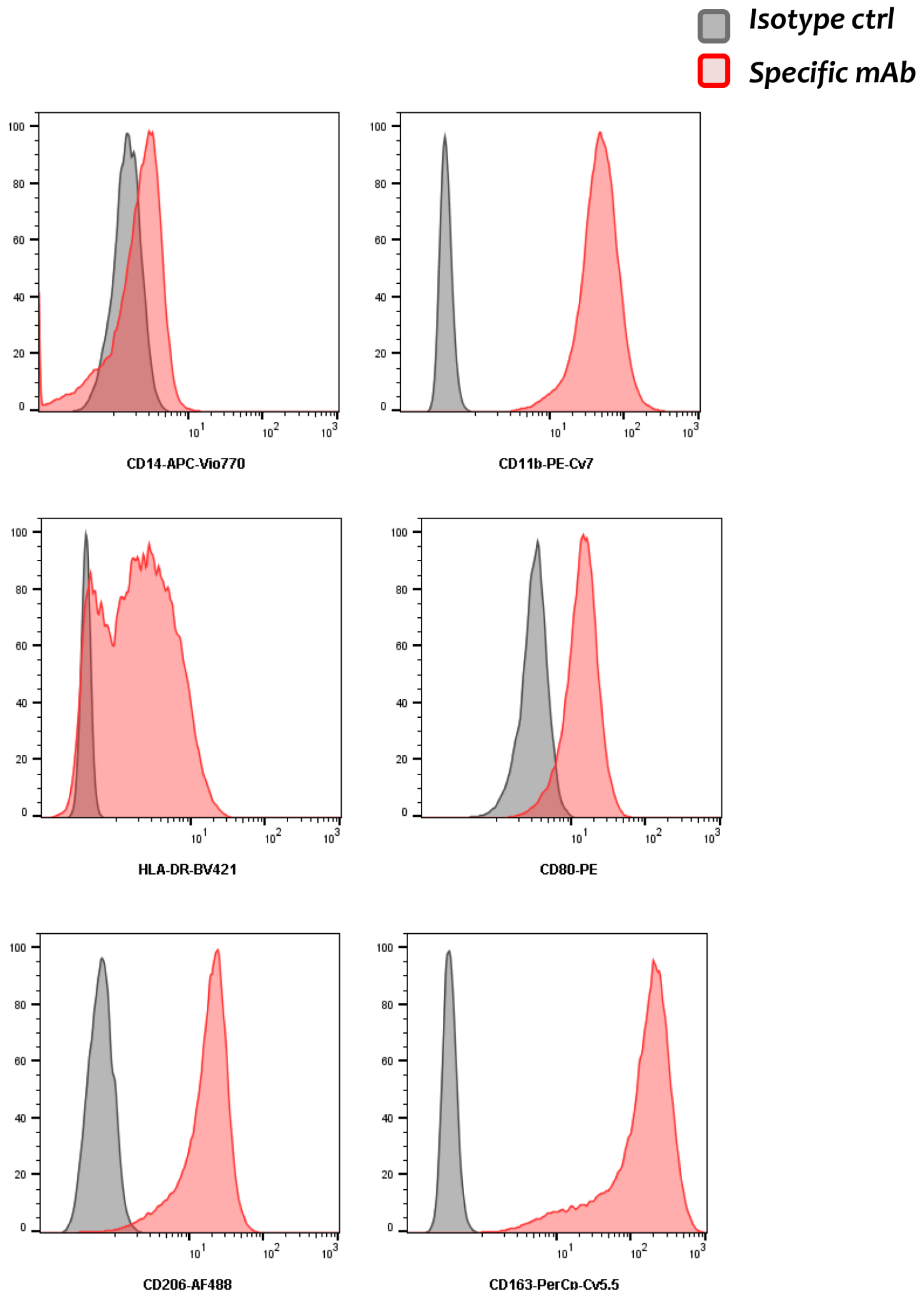

Supplementary Figure 6: In tumor cell killing assays, 3C23K does not alter the numbers of macrophages. (A) (A and B) Quantification of CD14+ (A) and CD206/CD163+ **(B)** macrophages at day 1 and day 6 of mAb treatment with either the irrelevant mAb R565 (isotype Ctrl), the anti-AMHRII FcKO or the anti-AMHRII 3C23K. Data are expressed as total CD14+ or CD206+/CD163+ macrophages percentage +/- Standard Deviation (triplicates).

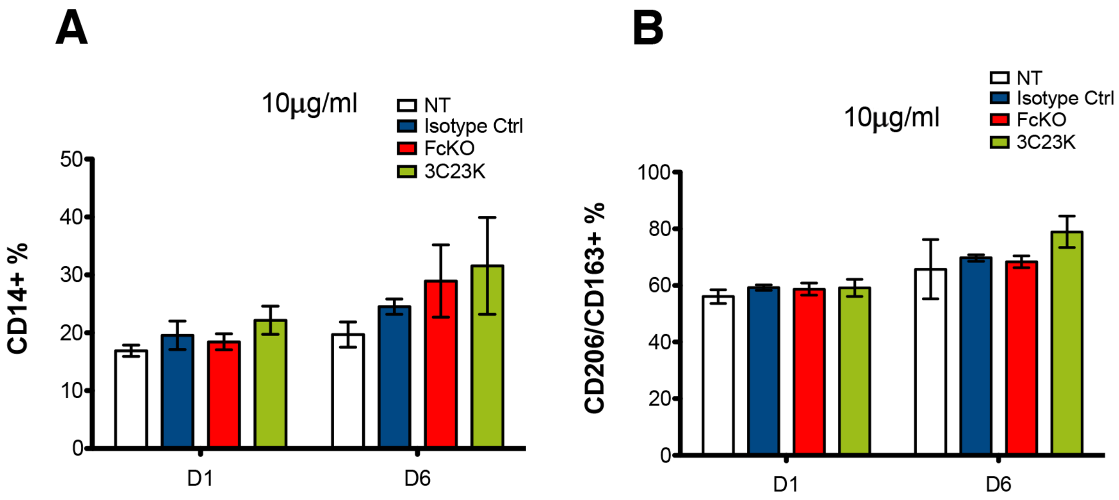

Supplementary Figure 7: 3C23K antibody does not affect the proliferation of human T lymphocytes. CellTrace Violet loaded T cells were activated byCD3/CD28 coated beads in presence or absence of 10 µg/ml of the anti-AMHRII 3C23K mAb. 3 days later the dilution of CellTrace Violet was evaluated by flow cytometry and represented as raw data **(A)** and as the % of T cells that have divided 1, 2, 3 or 4 times **(B)**.

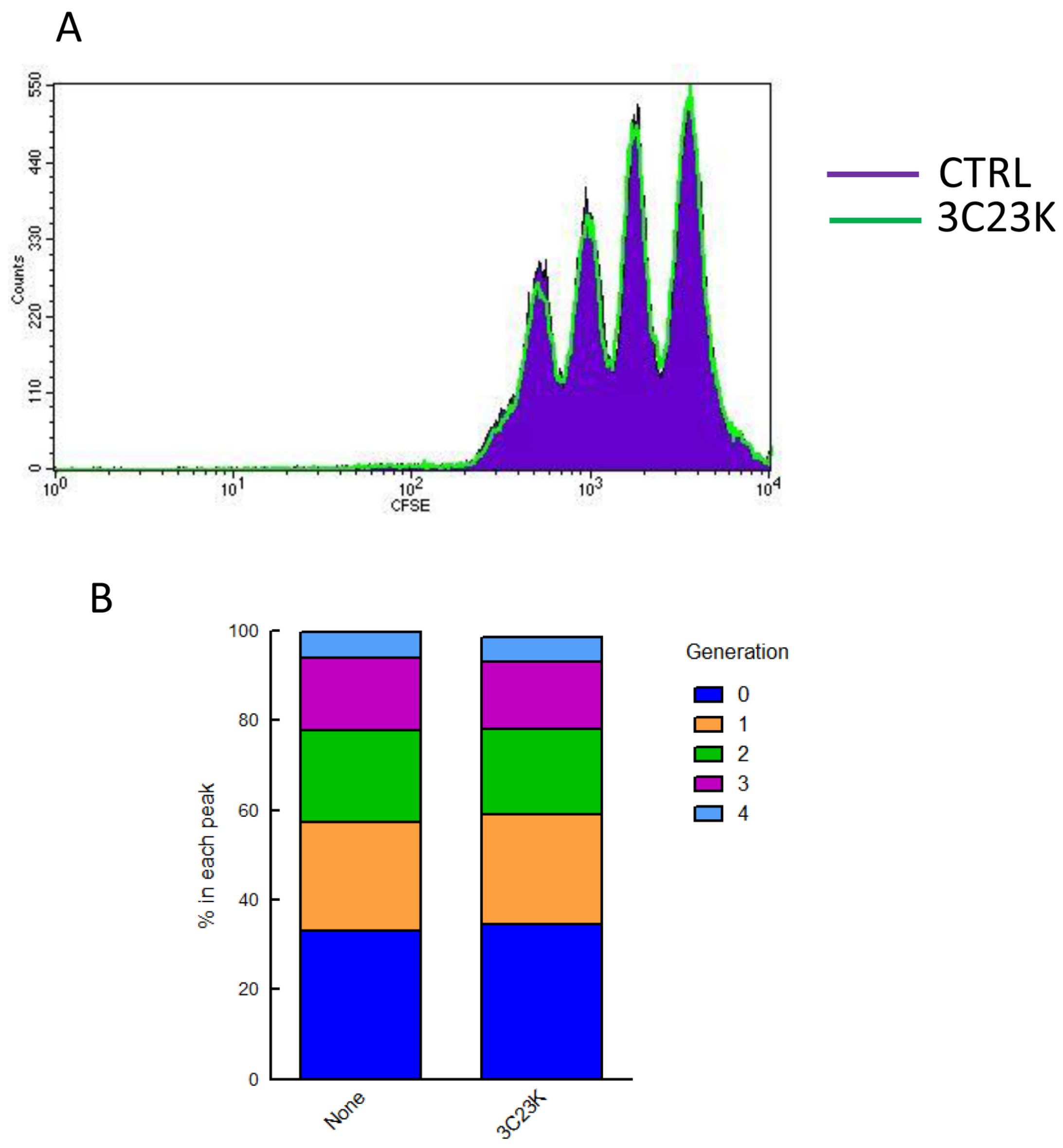

Supplementary Figure 8: Proposed model for the mode of action of 3C23K anti-AMHRII antibody. 3C23K binds to AMHRII expressed by ovarian tumor cells. Macrophages surrounding tumor cells engage 3C23K through their Fc receptors, and kill tumor cells. This leads to: i) ADCC and ADCP leading to tumor cell death, and ii) reduction of T cell suppression.

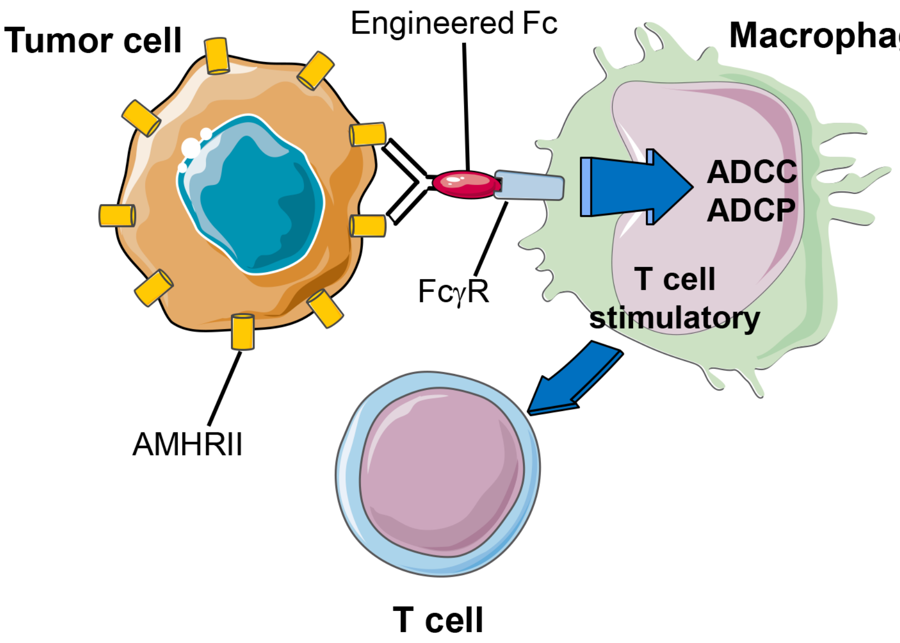

Supplementary Figure 9: Proposed model for the mode of action of 3C23K anti-AMHRII antibody. 3C23K binds to AMHRII expressed by ovarian tumor cells. Macrophages surrounding tumor cells engage 3C23K through their Fc receptors. This leads to: i) ADCC and ADCP leading to tumor cell death, and ii) reduction of T cell suppression.

A

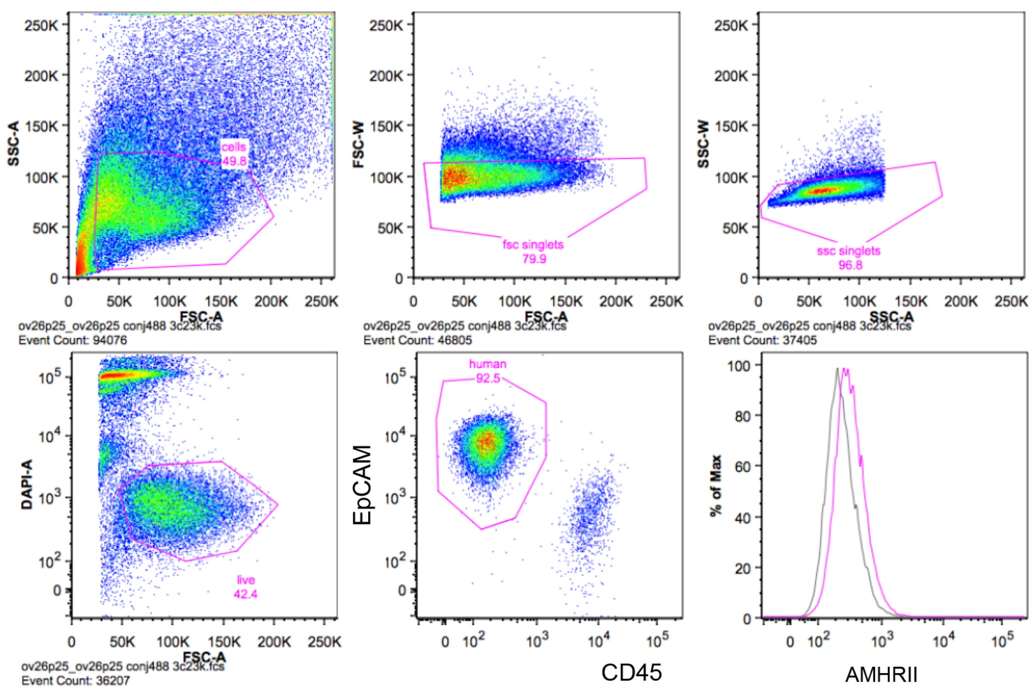

B

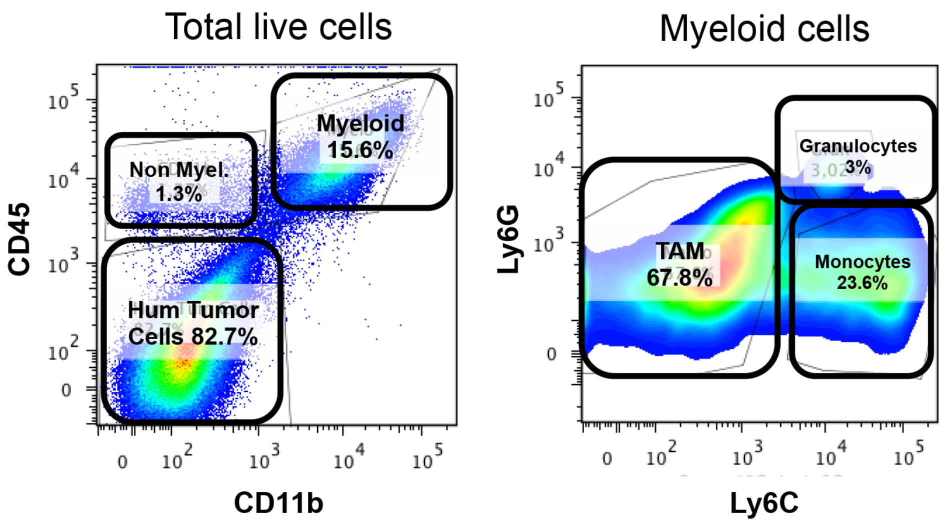

Supplementary Figure 10: FACS analyses. **(A)** Gating strategy of human tumor cells in PDX models. **(B)** Gating strategy of immune cells in human ovarian tumors.

Supplementary Table 1 : Ovarian Patient characteristics

Mean Age, y.	#	Tumor histology (n)	Tumor site	FIGO Tumor stage (n)	Chemotherapy (n)	AMHRII IF Score (n)	% of AMHRII + tumor cells by FACS (n)	AMHRII cell surface quantification
	49	Benign (1)	Ovary (1)	n.a. (1)	None (1)	-	n.a.	n.a.
	47<br>52	Borderline (3)	Ovary (2)	I A (2)	None (2)	++ (2)	29.50% (1)	42292<br>n.a.
	33		PC (1)	n.a. (1)	None (1)	+	n.a.	n.a.
	24	Clear Cell (1)	PC (1)	III C (1)	None (1)	-		n.a.
		6	Endometroid (1)	Ovary (1)	II A (1)	None (1)	+	n.a.
	39	MMMT (2)	Ovary (1)	II A (1)	None (1)	+++	6.3%	n.a.
		53	PC (1)	III C (1)	Yes (1)	- (2)	n.a.	n.a.
		34	Serous (24)	Lymp Node (1)	III A1(1)	None (1)		n.a.
		42	Ovary (6)	n.a. (1)	None (1)	+ (2)	1.3% (1)	n.a.
		18			II A (1)	None (1)		n.a.
		4		III A1 (1)	None (1)	++	n.a.	n.a.
		56		III C (2)	None (2)	+		n.a.
		50			++			n.a.
		8			IV B (1)	None (1)	+++	n.a.
		44		PN (1)	II B (1)	None (1)	-	n.a.
61		36	PC (15)	II B (1)	None (1)	+	22%	n.a.
		27	III B (5)		None (4)	-	0%	n.a.
		38				-	n.a.	n.a.
		29					+	n.a.
		51					++	n.a.
		22				Yes (1)	-	n.a.
		5		III C (6)	None (1)	++	29.5%	n.a.
		40			Yes (5)	+	11.0%	n.a.
		41				+	7,1%	n.a.
		45				++	9%	168000
		26				n.a.	70.0%	n.a.
		35				+	n.a.	n.a.
		31		IV A (2)	None (2)	++	13.3%	n.a.
		7				++	15.7%	n.a.
		37		IV B (1)	None (1)	++	n.a.	n.a.
	30		Pleural Biopsy (1)	IV A (1)	None (1)	+	n.a.	n.a.
n.a.	46	n.a. (2)	n.a.	n.a.	n.a.	-	0%	350
n.a.	43					++	na	n.a.
			**n (34)**	**IF positive AMHRII frequency**	**70.6%**			

Abbreviations: MMMT, Malignant Mixed Müllerian Tumor; PC, Peritoneal Carcinomatosis; PN, Pararectal Nodule; n.a., Not available; IF, ImmunoFluorescence.

Supplementary Table 2: Affinity constants (K_D) of 3C23K for the different humanand murine Fcγ receptors

Receptor	Human	murine
Fcγ RI/CD64	0.2-3.3**	15*
Fcγ RII/CD32a	120*	-
Fcγ RIIbc/CD32bc	459*	1106*
Fcγ RIIIa/CD16a	1.3-46**	-
Fcγ RIIIb/hCD16b	49*	1141*
Fcγ RIV/mCD16-2	-	2.1-49**

Affinity constants are expressed as KD in nM.
* KDwas calculated by using the heterogeneous ligand fitting model.
** KD values were determined with the two state reaction model when the fitting did not adhere to the heterogeneous ligand model.

Supplementary Table 3: ***In vivo*** **efficacy of 3C23K alone, chemotherapy alone and 3C23K + chemotherapy**

PDXs	Treatments	N of mice	TGI (%)	N (%) of CR	Median CR duration (days)	Median 1st day of CR
**OV16**	3C23K	8	0	0 (0)	/	/
	CT	8	95	3 (37)	25	37
	3C23K + CT	9	97	6 (67)	14	37
**OV21**	3C23K	8	23	0 (0)	/	/
	CT	9	80	0 (0)	/	/
	3C23K + CT	8	89	1 (12)	/	/
**OV25**	3C23K	10	35	1 (10)	/	/
	CT	10	91	1 (10)	/	/
	3C23K + CT	10	82	3 (30)	73	135
**OV42**	3C23K	8	0	0 (0)	/	/
	CT	8	83	1 (12)	/	/
	3C23K + CT	8	78	1 (12)	/	/
**OV54**	3C23K	10	25	0 (0)	/	/
	CT	10	86	1 (10)	/	/
	3C23K + CT	10	98	6 (60)	15	9

Abbreviations: N, number; TGI, Tumor Growth Inhibition; CR, complete remission, CT, chemotherapy included carboplatin + paclitaxel.

Supplementary Table 4: Histopathology of patient's tumors and corresponding PDXs

Tumeur	Patient Histology	PDX Histology	mutations
**OV16**	Serous adenocarcinoma moderately differentiated	Serous adenocarcinoma moderately differentiated	/
**OV21**	Serous papillary adenocarcinoma and mucinous (major component)	Serous papillary adenocarcinoma and mucinous (major component)	/
**OV25**	Serous adenocarcinoma moderately differentiated	Serous adenocarcinoma moderately differentiated	*BRCA1*
**OV42**	Heterogeneous carcinosarcoma with cartilaginous component and clear cells	Heterogeneous carcinosarcoma with osteoid component	/
**OV54**	Poorly differentiated serous adenocarcinoma	Undifferenciated serous adenocarcinoma	/
